# Computational Analysis of Transcriptomic and Proteomic Data for Deciphering Molecular Heterogeneity and Drug Responsiveness in Model Human Hepatocellular Carcinoma Cell Lines

**DOI:** 10.3390/genes11060623

**Published:** 2020-06-05

**Authors:** Panagiotis C. Agioutantis, Heleni Loutrari, Fragiskos N. Kolisis

**Affiliations:** 1 Biotechnology Laboratory, School of Chemical Engineering, National Technical University of Athens, 5 Iroon Polytechniou Str., Zografou Campus, 15780 Athens, Greece; panagiout@mail.ntua.gr; 2 G.P. Livanos and M. Simou Laboratories, 1st Department of Critical Care Medicine & Pulmonary Services, Evangelismos Hospital, Medical School, National Kapodistrian University of Athens, 3 Ploutarchou Str., 10675 Athens, Greece

**Keywords:** hepatocellular carcinoma, transcriptomics, proteomics, bioinformatics analysis, differentiation, Gene Ontology, Reactome Pathways, gene-set enrichment

## Abstract

Hepatocellular carcinoma (HCC) is associated with high mortality due to its inherent heterogeneity, aggressiveness, and limited therapeutic regimes. Herein, we analyzed 21 human HCC cell lines (HCC lines) to explore intertumor molecular diversity and pertinent drug sensitivity. We used an integrative computational approach based on exploratory and single-sample gene-set enrichment analysis of transcriptome and proteome data from the Cancer Cell Line Encyclopedia, followed by correlation analysis of drug-screening data from the Cancer Therapeutics Response Portal with curated gene-set enrichment scores. Acquired results classified HCC lines into two groups, a poorly and a well-differentiated group, displaying lower/higher enrichment scores in a “Specifically Upregulated in Liver” gene-set, respectively. Hierarchical clustering based on a published epithelial–mesenchymal transition gene expression signature further supported this stratification. Between-group comparisons of gene and protein expression unveiled distinctive patterns, whereas downstream functional analysis significantly associated differentially expressed genes with crucial cancer-related biological processes/pathways and revealed concrete driver-gene signatures. Finally, correlation analysis highlighted a diverse effectiveness of specific drugs against poorly compared to well-differentiated HCC lines, possibly applicable in clinical research with patients with analogous characteristics. Overall, this study expanded the knowledge on the molecular profiles, differentiation status, and drug responsiveness of HCC lines, and proposes a cost-effective computational approach to precision anti-HCC therapies.

## 1. Introduction

Liver cancer is one of the most frequently occurring life-threatening neoplasms worldwide. Hepatocellular carcinoma (HCC)—the most predominant type of primary liver cancer (85% to 90%)—usually arises in the context of inflammatory-induced stress and chronically progressing liver cirrhosis. Depending on the region of incidence, a wide range of risk factors have been implicated in the development of HCC. Hepatitis B and aflatoxin B1 exposure are widely associated with HCC cases occurring in eastern Asia and Sub-Saharan Africa, while hepatitis C, alcohol consumption, and non-alcoholic fatty liver disease prevail as leading causative factors in the Western world and Japan [1]. Regardless of the potentially implicated risk factors, HCC incidence constitutes a remarkably complex multistep process, involving various genomic and epigenomic aberrations leading to an inevitable molecular heterogeneity [2,3]. Deregulated cell proliferation, increased inflammatory and oxidative stress, enriched tumor microenvironment, and abnormally active angiogenic switches characterize the progression of HCC, through the early stage of initiation up to the point of invasion and metastasis [4]. 

Despite existing surveillance protocols for cirrhotic patients, HCC is often diagnosed in advanced stages, resulting in limited applicable treatments or effective therapies. Moreover, HCC chemopreventive strategies are additionally hampered by the aforementioned innate molecular heterogeneity of the disease. To date, only a handful of available treatment regimens have been proven effective (first-line options sorafenib and lenvatinib, along with second-line options regorafenib, cabozantinib, and ramucirumab), and only to some extent [4]. Therefore, it is of the utmost importance to identify novel biomarkers and potent drug agents to address the miscellaneous characteristics of HCC cases. Cancer cell lines have been extensively used during the last decades as valuable research model systems. Although not ideal in portraying the physiological and molecular traits of patients’ malignancies, they have enabled a plethora of low-cost experiments towards the genomic and functional characterization of cancers [5]. Furthermore, diverse well-characterized cell lines could provide a convenient platform for pharmacogenomic studies deciphering the molecular complexity of tumors in association with drug-specific sensitivity [6,7].

To this end, in the present work, we aimed to shed light on the distinct/shared molecular features of 21 widely used human HCC cell lines (HCC lines), and to investigate their potential connection to drug efficiency. Our strategy implemented a thorough bioinformatics exploratory and functional enrichment analysis of publicly available transcriptomic and proteomic data from HCC lines, along with a correlation analysis of existing drug-response data to defined molecular signatures. Furthermore, tumors from HCC patients were characterized accordingly by analyzing gene expression data from the Cancer Genome Atlas (TCGA). Acquired results provided information on (a) potentially discrete subtypes amongst the investigated HCC lines, mainly dictated by their molecular resemblance (or not) to normal hepatocytes; (b) differentially expressed genes (DEGs) and differentially expressed proteins (DEPs) representative of inherent cancer heterogeneity; (c) biological processes and pathways significantly related to DEGs; and (d) HCC-subtype-specific sensitivity/resistance to drugs. Overall, the present work sets the basis of a computational platform for the integration and analysis of publicly accessible -omics and drug-screening data from tumor cell lines—and eventually tissue specimens—enabling the development of patient-tailored anti-cancer medications.

## 2. Materials and Methods

### 2.1. Data Acquisition and Pre-Processing

Publicly available transcriptomic and proteomic data were obtained from the Cancer Cell Line Encyclopedia (CCLE) database [7] (https://portals.broadinstitute.org/ccle/data). Gene-centric RMA-normalized mRNA expression (CCLE_Expression_Entrez_2012-09-29.gct), reverse-phase protein array (RPPA-CCLE_RPPA_20181003.csv), and HCC line annotation (CCLE_sample_info_file_2012-10-18.txt) data were downloaded. The RPPA data file contained median-centered log_2_-normalized relative protein expression values as previously described [8]. Out of all the total cancer cell line entries in the CCLE database, 23 liver cancer cell lines with available microarray gene expression data and RPPA protein expression data were extracted. Two cell lines—namely SNU398 and NCIH684—were excluded from subsequent analyses, the former due to the highly anaplastic nature of the cells [9], and the latter because it originates from primary colon cancer metastasized to the liver. Remaining liver cancer cell lines included 20 HCC lines and SKHEP1, a widely used liver adenocarcinoma cell line of endothelial origin [10]. SKHEP1 has been sporadically used in HCC-associated studies, despite recent recommendations [11].

Drug-screening data containing area under concentration–response curve (AUC) sensitivity measurements for 481 drugs, regarding 15 of the extracted cell lines, were obtained from the Cancer Therapeutics Response Portal (CTRPv2, https://portals.broadinstitute.org/ctrp.v2.1/) [12]. AUCs are based on percentage viability scores compared to dimethyl sulfoxide (DMSO)-treated cells [13]. Drugs with missing values for three or more cell lines were subsequently discarded. To conclude, 21 HCC lines (Table 1) with corresponding gene expression (18,900 genes) and protein/phosphoprotein expression (214 proteins/phosphoproteins) data were studied. For the vast majority of cell lines, additional drug-screening sensitivity data for 344 compounds were investigated.

Finally, gene-expression RNA-seq data from the TCGA HCC cohort [14] were downloaded as gene-level raw expression values produced by *RSEM* [15] (LIHC.uncv2.mRNAseq_raw_counts.txt) from the Broad Institute portal (https://gdac.broadinstitute.org/) along with corresponding clinical information. Raw gene expression values were appropriately normalized using the TMM (trimmed mean of M values) normalization method [16] and transformed in log_2_ scale.

### 2.2. Exploratory Analysis of Transcriptomic and Proteomic Data

Pairwise Pearson’s correlation coefficients were computed between each pair of HCC lines, based on the expression of the 500 genes with the largest cross-sample variation (median absolute deviation) and the expression of 214 available proteins/phosphoproteins, respectively. Graphical displays of correlation matrices were produced using the *corrplot* package in R. 

Principal component analysis (PCA) was performed using the dedicated PCA function from the *mixomics* R package [17]. Optimal univariate *k*-means clustering was conducted by implementing the *Ckmeans.1d.dp* package in R [18]. The core function of this package performs one-dimensional (1D), weighted or unweighted, *k*-means clustering and provides the optimal number of clusters using the Bayesian information criterion (BIC) [19]. HCC line weights were considered equal (weight = 1.0). 

Single-sample gene-set enrichment analysis (ssGSEA) scores were computed against curated gene-sets (C2) from MSigDB by implementing the *GSVA* package in the R environment [20]. ssGSEA defines an enrichment score that represents the degree of absolute enrichment of a gene-set in each sample within a given dataset [21]. Essentially, ssGSEA enrichment scores signify the degree to which genes in a particular gene-set are coordinately up- or downregulated within a given sample.

A recently published epithelial-to-mesenchymal transition (EMT) gene expression signature [22] consisting of 239 genes —215 epithelial and 24 mesenchymal markers— was further used to enhance the exploratory data-analysis process. More specifically, hierarchical clustering (average linkage, Euclidean distance) was performed based on the EMT signature, to support/supplement PCA-identified clusters.

### 2.3. Between-Group Differential Gene and Protein Expression Analysis

Between-group gene and protein differential expression analyses were conducted by implementing the *limma* package in R [23]. Genes with overall very low expression were filtered out, while the full set of available proteins/phosphoproteins was used. Regarding the identification of DEGs, the *treat* function [24], which tests for significance relative to fold-change thresholds, was implemented. Genes with an adjusted *p*-value < 0.1 (Benjamini–Hochberg correction) and |fold-change| > 1.2 were considered DEGs. Proteins/phosphoproteins with an adjusted *p*-value < 0.1 (Benjamini–Hochberg correction) after moderated *t*-tests implemented through the *eBayes* function, were considered differentially expressed as well. Volcano plots illustrating identified DEGs and DEPs were created using the *EnhancedVolcano* package in R [25]. Scaled gene/protein expression values were used in heatmap illustrations for individual HCC lines regarding identified DEGs and DEPs. 

### 2.4. Functional Enrichment Analysis of Differentially Expressed Genes

Reactome Pathway and Gene Ontology (GO) enrichment analysis of DEGs was conducted using *Bioinfominer* [26,27], a bioinformatics tool that delivers unsupervised, fast, and integrative interpretation of -omics experiments. This tool accepts lists of genes and performs enrichment analysis along with prioritization of detected systemic processes, ultimately resulting in a compact signature consisting of systemic processes and their hub driver-genes. This signature constitutes a deconvoluted projection onto biological networks of hierarchical structure (ontologies, Reactome Pathway database), corrected for biases as well as other inconsistencies. The significance threshold for altered biological processes/pathways was set at a corrected hypergeometric *p*-value of 0.05. 

Testing for enrichment of curated gene-sets (C2) from MSigDB amongst the between-group differential gene expression data was performed using the *camera* function [28] in the *limma* package, a competitive gene-set test procedure based on the idea of taking into account the intergene correlation to adjust the gene-set test statistic. Statistically significant enriched gene-sets were controlled at an adjusted FDR = 0.05 threshold, after Benjamini–Hochberg correction for multiple testing. 

### 2.5. Drug-Specific Sensitivity in Association with Differentiation Status of HCC lines

Drug-sensitivity AUC measurements available for 15 of the HCC lines (Table 1, blue font) were correlated with their enrichment scores for a specific/selected (SU_LIVER) gene-set, in an attempt to elucidate differentiation-status-associated drug-sensitivity. Lower AUC sensitivity measurements corresponded to an enhanced drug effect against cell line viability. Liver-like well-differentiated cell lines were characterized by higher enrichment scores than the poorly differentiated ones; therefore, positive correlations highlighted drugs more effective against poorly differentiated cell lines, while negative correlations drugs more effective against well-differentiated ones. Drugs with a *p*-value < 0.05, an adjusted *p*-value < 0.3 (after Benjamini–Hochberg correction), and |Spearman’s ρ| > 0.5 were considered to be significantly correlated with the investigated enrichment score.

### 2.6. HCC Tumor Clustering Based on SU_LIVER Gene-Set Expression Data From TCGA 

HCC patients (*n* = 354) with available gene-expression data and documented histological grades were extracted from the downloaded TCGA RNA-seq dataset. A total 224 samples have been characterized as well/moderately differentiated tumors of Grade 1 and Grade 2 (G1+G2), while 130 samples have been characterized as poorly/undifferentiated tumors of Grade 3 and Grade 4 (G3+G4). Hierarchical clustering (Ward linkage, Euclidean distance) of the 354 patients was conducted based on the scaled expression of all 58 genes involved in the SU_LIVER gene-set. Subsequently, a χ^2^ test in the R environment was conducted to test for a possible association between the formed clusters and histological grading. 

## 3. Results

### 3.1. HCC Lines Clustered into Two Distinct Differentiation Subtypes

**Figure 1:**
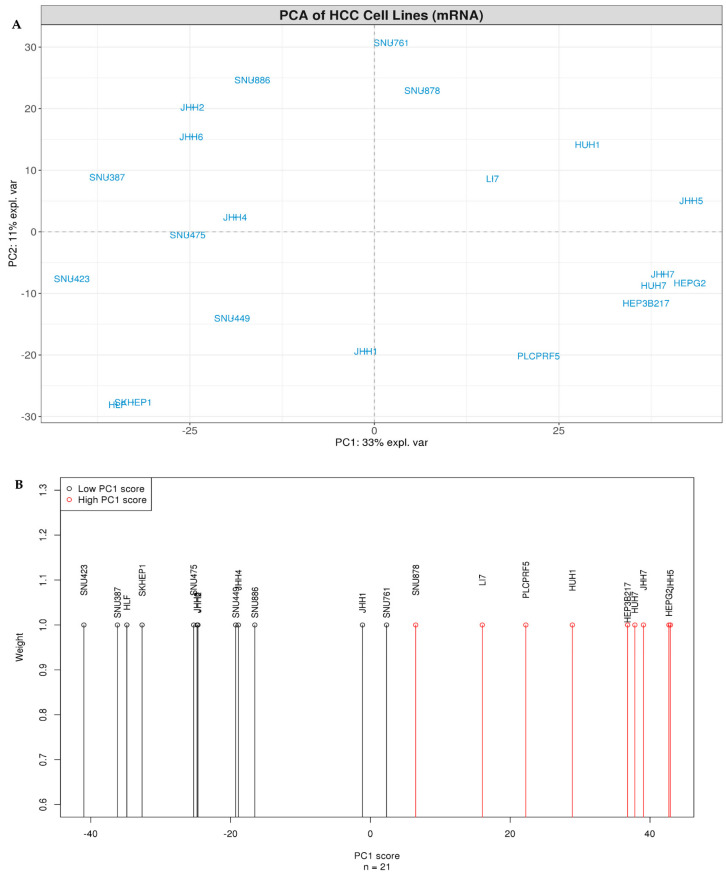
Gene-based principal component analysis (PCA) and clustering of HCC lines (**A**) PCA on 500 genes with the largest cross-sample variation. PC1 (x-axis) versus PC2 (y-axis) for 21 HCC lines indicated by blue color. Dashed horizontal and vertical lines mark zero values of PC1 and PC2, respectively. (**B**) The two optimal *k*-means clusters based on PC1 scores as identified by the Bayesian information criterion (BIC). All HCC lines were treated as equally weighted.

The present study focused on the global comparison of 21 patient-derived HCC lines for which gene and protein/phosphoprotein expression data are publicly available (Table 1). An initial correlation matrix analysis based on (i) the expression of 500 genes exhibiting the largest cross-sample variation (Figure S1) and (ii) the abundance of all 214 proteins/phosphoproteins offered from the RPPA dataset (Figure S2) notably showed that some of the examined cell lines shared a higher similarity compared to others. Further investigation of these results by PCA, using the same subset of 500 genes, revealed a clear spread of HCC lines across PC1, explaining 33% of variance amongst samples (Figure 1A). Subsequent 1D *k*-means clustering performed on the PC1 scores of HCC lines indicated two optimal discrete clusters across PC1 (Figure 1B, Figure S3) which notably corresponded to a “high PC1 score” cluster and a “low PC1 score” cluster, respectively. 

Compared to the microarray gene expression data, RNA-seq data retrieved from CCLE provided almost identical results for the examined HCC lines in gene-based PCA (Figure S4). Most importantly, a high cross-platform Pearson correlation (mean value 0.85 ± 0.01 standard deviation) was found for all 21 same-cell-line pairs (e.g., HEPG2_RNAseq_ – HEPG2_microarray_), based on the expression of 16,667 genes found to be shared within the two datasets.

**Figure 2:**
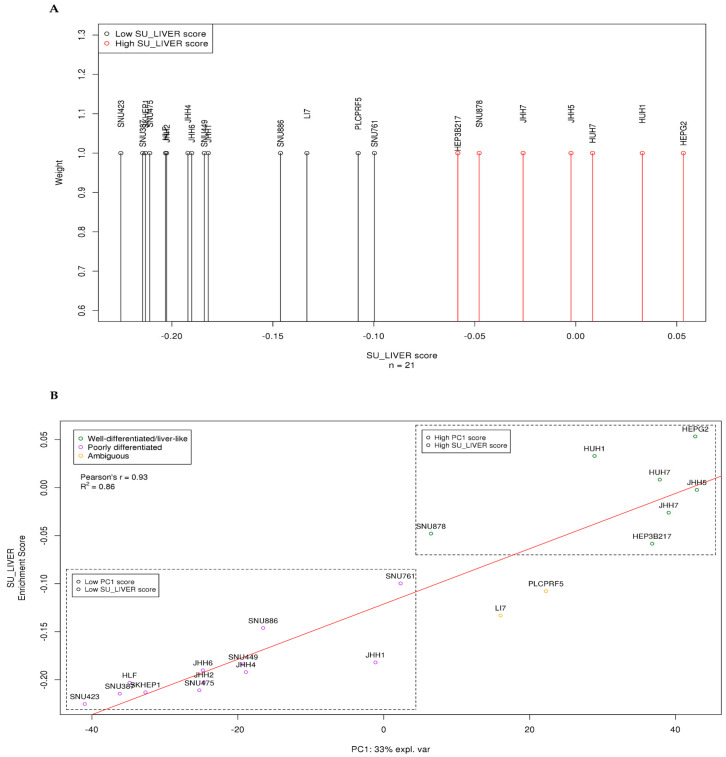
Specifically Upregulated in Liver (SU_LIVER) clustering of HCC lines and PC1-SU_LIVER correlation. (**A**) The two optimal *k*-means clusters based on computed cell line ssGSEA SU_LIVER enrichment scores as identified by the BIC. All HCC lines were treated as equally weighted. (**B**) PC1 score correlation (Pearson’s) with individual cell-line enrichment scores for the SU_LIVER gene-set. Cell lines included in the “high SU_LIVER score”/“high PC1 score” clusters were identified as liver-like and well-differentiated (green circles), while the ones in the “low SU_LIVER score”/“low PC1 score” clusters were characterized as poorly differentiated (purple circles). Yellow circles indicate ambiguous cell lines.

In order to highlight the biological background underlying this discrete cell-line grouping across PC1, we subsequently computed the ssGSEA scores against curated gene-sets from MSigDB, based on calculated PC1 loadings (Table S1). Interestingly, a “Specifically Upregulated in Liver” gene-set (SU_LIVER), containing genes upregulated specifically in human liver tissue [29], was identified as one of the top gene-sets with positive enrichment scores. Additionally, individual cell-line enrichment scores for each particular gene-set were computed by ssGSEA (Table S2). After examining and evaluating many HCC-related top enriched gene-sets regarding their ability to predict PC1 scores, we focused on SU_LIVER, as this gene-set exhibited the best predictive performance. We performed 1D *k*-means clustering, which again highlighted two optimal clusters (Figure 2A, Figure S5), a “high SU_LIVER score” and a “low SU_LIVER score” cluster, respectively. Subsequent correlation of PC1 scores with the individual cell-line SU_LIVER enrichment scores revealed that 86% of observed PC1 variance was explained by that term (Pearson’s *r* = 0.93, *R*^2^ = 0.86, *p*-value = 1.874 × 10^–9^). Cell lines included in both the “high SU_LIVER score” and “high PC1 score” clusters were therefore considered to be liver-like and well-differentiated, while the ones belonging in both the “low SU_LIVER score” and “low PC1 score” clusters were characterized as poorly differentiated (Figure 2B). 

Results for only two cell lines, namely LI7 and PLCPRF5, were conflicting, because their PC1 score clustering opposed their SU_LIVER enrichment score clustering; therefore, these cell lines were characterized as ambiguous.

**Figure 3:**
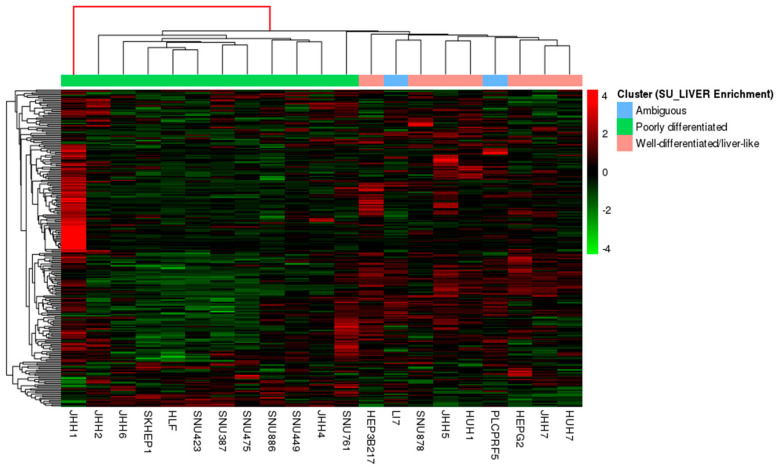
Heatmap illustrating the hierarchical clustering of cancer cell lines (columns) based on the epithelial-to-mesenchymal transition (EMT) signature of 239 genes (rows). Scaled values indicate relative downregulation (green color) or upregulation (red color) of gene expression. Cell lines are annotated by color, based on the clusters that were predicted by the SU_LIVER enrichment scores shown in Figure 2B.

We subsequently used a recently published EMT gene expression signature to further challenge the proposed differentiation-associated stratification of test HCC lines, since EMT is a process highly related to the differentiation/de-differentiation status of cancer cells [29]. Notably, the hierarchical clustering of HCC lines —as depicted in the corresponding heatmap— unveiled again their classification into two main groups, based on the EMT gene expression signature pattern, while additionally identified JHH1 as a rather ambiguous cell line of discrete nature (Figure 3). This clustering generally corroborated the differentiation groups demonstrated in Figure 2B.

**Figure 4:**
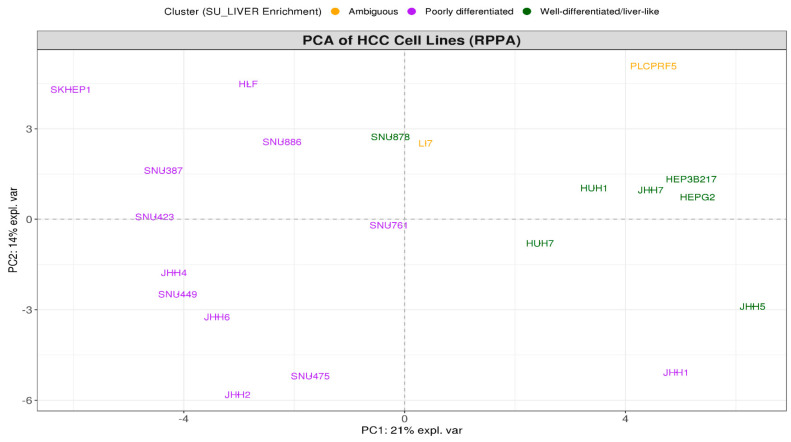
PCA based on all 214 protein/phosphoprotein reverse-phase protein array (RPPA) expression data. PC1 (x-axis) versus PC2 (y-axis). Cell lines are annotated by color, based on the gene-expression-derived clusters that are predicted by the SU_LIVER enrichment scores shown in Figure 2B.

Finally, PCA based on protein/phosphoprotein RPPA expression data showed that the clustering of HCC lines was widely consistent, not only at the gene but also at the protein expression level (Figure 4) and further confirmed the discrete/ambiguous nature of the JHH1 cell line. Based on these findings, JHH1 along with LI7 and PLCPRF5 were considered to be ambiguously characterized in the context of this study and were therefore excluded from downstream analyses, in order to get a more straightforward and comprehensive grasp of distinct differentiation-associated molecular characteristics amongst the remaining HCC lines.

### 3.2. Differential Gene and Protein Expression between Poorly and Well-Differentiated HCC Lines

**Figure 5:**
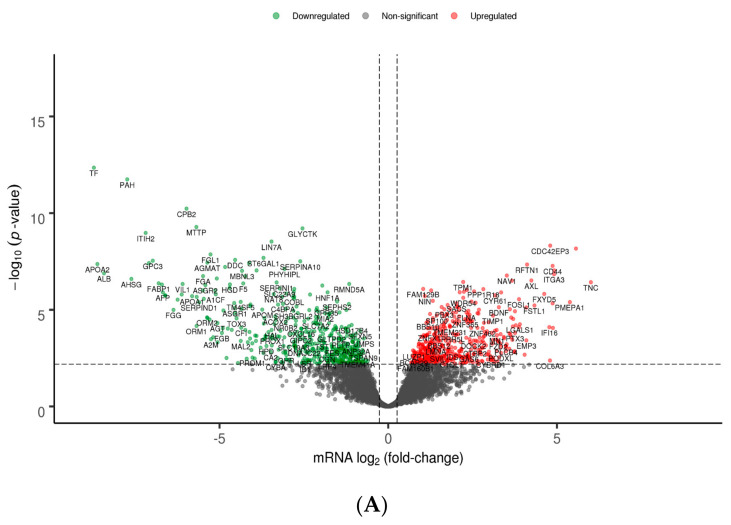

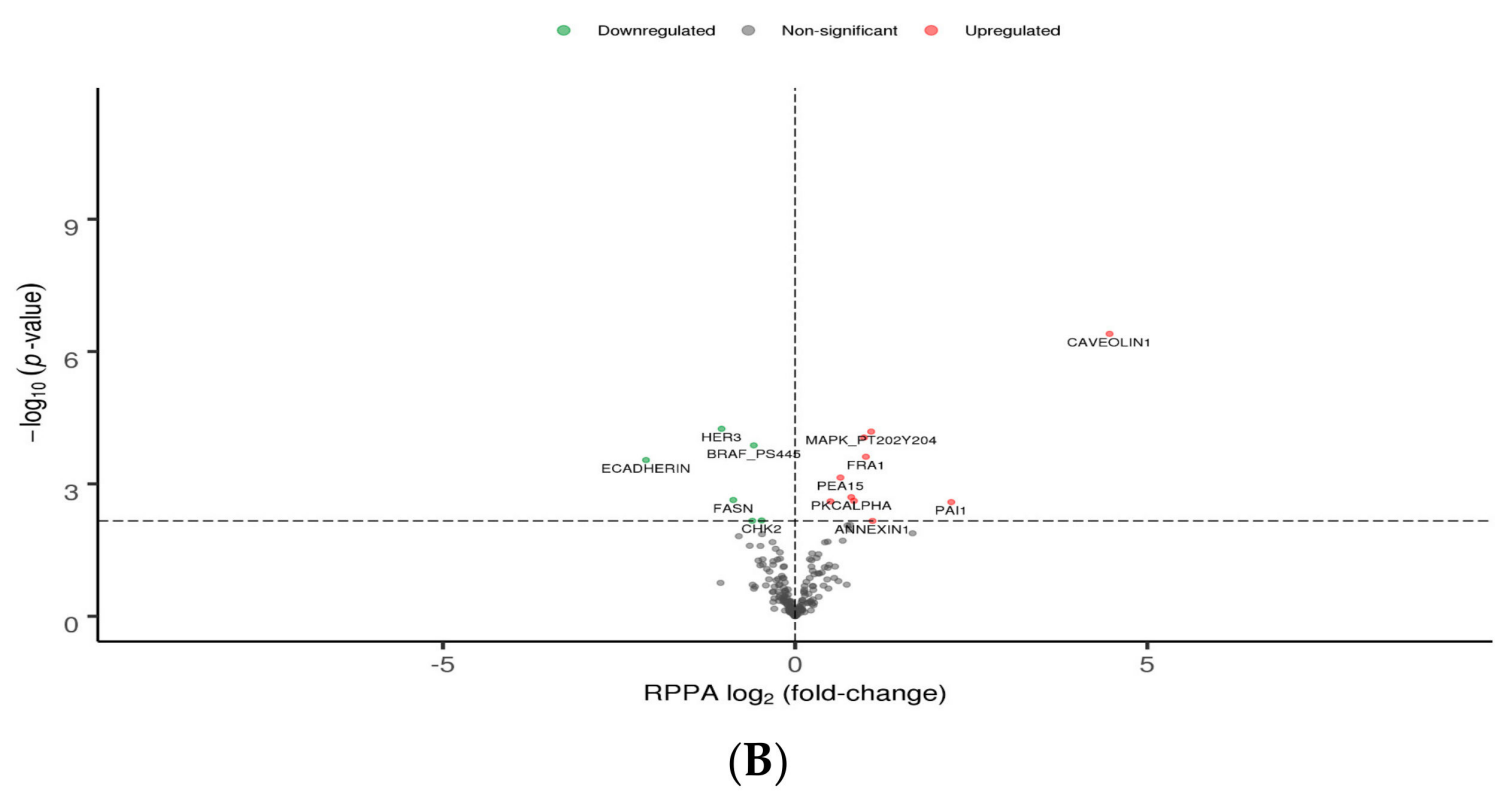
Volcano plots illustrating differentially expressed genes (DEGs) (**A**) and differentially expressed proteins (DEPs) (**B**) in poorly differentiated versus well-differentiated HCC lines. Red and green dots represent up- and downregulated genes/proteins, respectively; grey dots represent non-statistically-significant altered genes/proteins. Horizontal dashed lines indicate a statistical threshold corresponding to an adjusted *p*-value of < 0.1; x-axis: mRNA log_2_ fold-change (**A**) or RPPA log_2_ fold-change (**B**), y-axis: *p*-value in negative log_10_ scale.

Between-group differential gene and protein expression analysis was subsequently carried out to investigate the molecular basis underlying the distinct classification of the examined HCC lines. The volcano plots shown in Figure 5A,B depict the number of DEGs and DEPs respectively, between poorly and well-differentiated liver-like HCC lines. Considering the latter as controls, since they undoubtedly uphold molecular features closer to functional normal hepatocytes, we accordingly identified a significant differential expression of 935 genes (462 upregulated and 473 downregulated) and 16 proteins (10 upregulated and 6 downregulated). Full lists of DEGs and DEPs are provided in Tables S3 and S4. Additionally, heatmaps illustrating the scaled expression values of HCC lines regarding identified DEGs and DEPs are provided in Figures S6 and S7, respectively, offering a comprehensive representation of individual cell-line expression patterns irrespective of assigned control group. 

**Figure 6:**
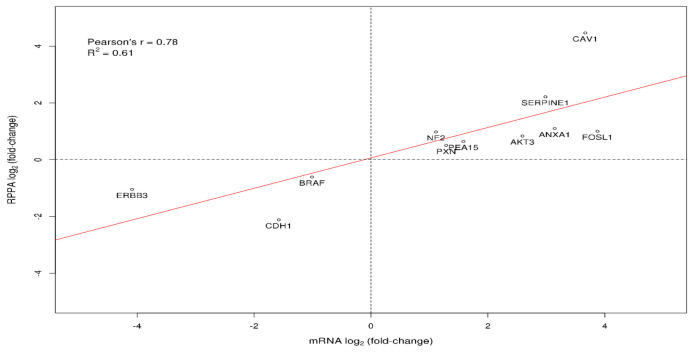
Pairwise Pearson correlation between identified DEPs (total proteins) and their corresponding DEGs. x-axis: mRNA log_2_(fold-change) of DEGs, y-axis: RPPA log_2_(fold-change) of DEPs. Protein–gene pairs are represented by their corresponding HGNC gene symbol.

Identified DEPs included downregulated proteins ECADHERIN, HER3, FASN, BRAF, CHK2, and phospho-BRAF, along with upregulated proteins CAVEOLIN1, PAI1, PAXILLIN, PEA15, PKCALPHA, AKT, NF2, FRA1, ANNEXIN1, and phospho-MAPK1/MAPK3. Pairwise total protein–mRNA relationships were investigated, excluding only for this analysis the two phosphorylated DEPs (MAPK_PT202Y204 and BRAF_PS445). For the majority of observed DEPs (HER3, ECADHERIN, BRAF, NF2, PAXILLIN, AKT, ANNEXIN1, PEA15, PAI1, FRA1, CAVEOLIN1) corresponding genes (*ERBB3, CDH1, BRAF, NF2, PXN, AKT3, ANXA1, PEA15, SERPINE1, FOSL1, CAV1*) were also found to be differentially expressed. A noteworthy observation was made concerning AKT: out of the three genes encoding for the corresponding isoforms—namely *AKT1*, *AKT2*, and *AKT3* (all detected by a single antibody in applied RPPA procedures [8])—only the expression of *AKT3* was significantly upregulated. Furthermore, mRNA expression fold-changes positively correlated with the respective total protein expression changes (Figure 6, Pearson’s *r* = 0.78, *R*^2^ = 0.61, *p*-value = 0.00458). The genes encoding the remaining DEPs (*FASN*, *CHK2*, and *PKCALPHA*) were either not identified as DEGs based on the predefined criteria, or were not included in the starting list of available genes.

### 3.3. Differentially Enriched Biological Processes/Pathways and Hub Driver-Gene Signatures between Poorly and Well-Differentiated HCC Lines

**Figure 7:**
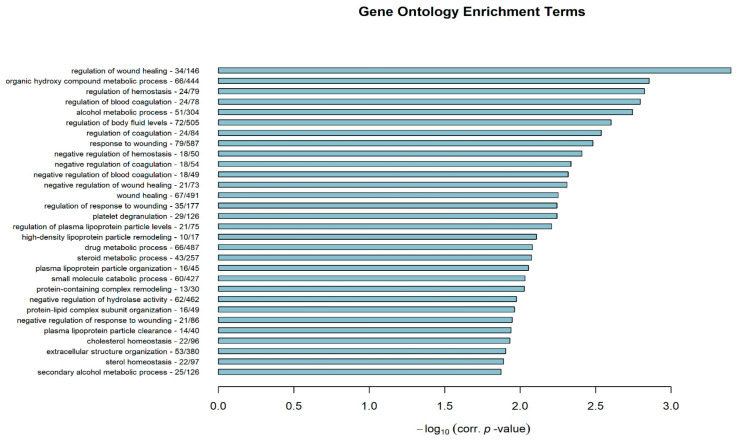
Top 30 significantly enriched Gene Ontology (GO) biological process terms, ranked by their hypergeometric corrected *p*-value in negative log_10_ scale (x-axis). Gene enrichment is also presented in total gene numbers, right after each GO term.

**Figure 8:**
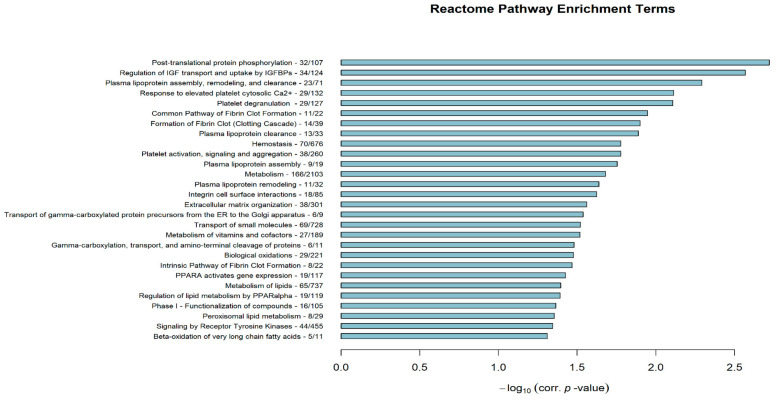
Significantly enriched Reactome Pathway terms ranked by their hypergeometric corrected *p*-value in negative log_10_ scale (x-axis). Gene enrichment is also presented in total gene numbers, right after each Reactome Pathway term.

To better comprehend the molecular basis underlying the classification of the examined HCC lines into two distinct differentiation subtypes, DEGs were next subjected to downstream functional GO and Reactome Pathway enrichment analysis to reveal potentially implicated biological processes/pathways. By implementing the *Bioinfominer* software, a total of 114 significantly enriched GO biological processes and 28 additional biological Reactome pathways were identified (Tables S5 and S6). Figure 7 depicts the top 30 enriched GO terms, ranked by their corrected hypergeometric *p*-values, while Figure 8 shows all Reactome-Pathway-enriched terms. Recurring biological features were easily identifiable in both enrichment datasets, as in fact terms associated to wound healing, blood coagulation and hemostasis, fibrinolysis and clotting cascades, extracellular matrix (ECM) organization, platelet activation, and cell migration and motility were commonly observed. Furthermore, terms related to altered metabolism along with lipid/cholesterol homeostasis were assertively present.

Apart from the typical enrichment analysis, we exploited the *Bioinfominer’s* ability to aggregate ontologically similar/interconnected enriched terms and prioritize them in the context of systemic processes for both GO and Reactome Pathway database vocabularies. A compact signature of hub driver-genes implicated in these prioritized systemic processes was produced in each case. As a result, systemic processes derived from the GO biological process and Reactome Pathway enrichment analyses were inferred and are presented in Figures S8 and S9, respectively. Prioritized systemic processes, as expected, included biological terms commonly encountered in both enrichment analyses, highlighting aforementioned recurring features such as fibrinolysis, hemostasis, platelet activation, wounding, ECM structure, and metabolism as core affected systemic processes, amongst others. Derived signatures of driver-genes associated with the recorded systemic processes are presented in Table 2 (41 hub genes, GO-based gene signature) and Table 3 (21 hub genes, Reactome-Pathway-based gene signature). 

**Figure 9:**
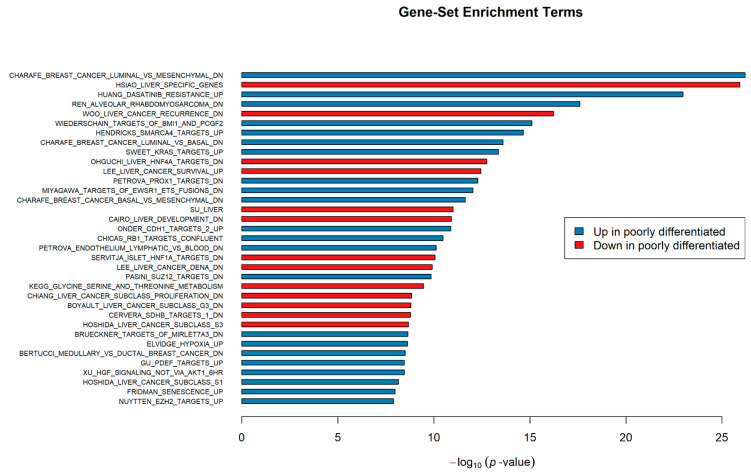
Top 35 gene-set enrichment terms as identified by *camera* testing, ranked by their *p*-value in negative log_10_ scale (x-axis).

Additional gene-set enrichment analysis using the R function *camera* [28] provided further complementary information about the differentiation-associated characteristics beyond the obtained GO and Reactome Pathway results, based on the differential expression analysis data between the two identified groups of HCC lines and against curated (C2) gene-sets from MSigDB. Complete gene-set enrichment results are provided in Table S7, and included an important number of statistically significant enriched gene-sets, along with the projected enrichment direction in each set (genes either up- or downregulated in poorly differentiated cell lines). Poorly differentiated HCC lines were characterized by downregulation of gene expression associated with epithelial liver-like traits (HSIAO_LIVER_SPECIFIC_GENES, SU_LIVER) [30,31] and various metabolic processes. In contrast, these HCC lines significantly overexpressed genes associated with Slug-related EMT initiation (ANASTASSIOU_CANCER_MESENCHYMAL_TRANSITION_SIGNATURE) [32], along with hypoxia (ELVIDGE_HYPOXIA_UP) [33] and migration (WU_CELL_MIGRATION) [34]. In addition, *camera* results unveiled a connection amongst HCC line subtypes and the three distinct molecular subclasses identified by Hoshida et al. [35] in HCC tissues (Subclasses S1, S2 and S3). The top 35 statistically significant gene-sets, ranked by their *p*-value, are illustrated in Figure 9. 

### 3.4. Cell Line Differentiation Status Correlated with Drug-Specific Sensitivity

**Figure 10:**
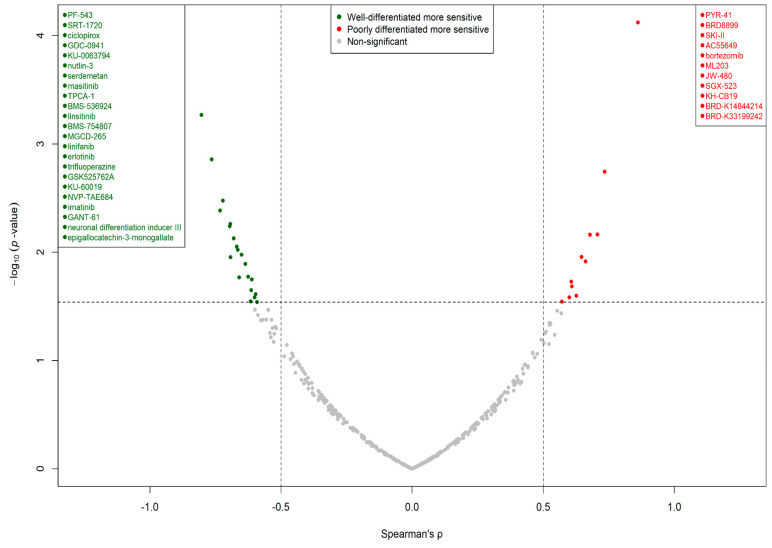
Volcano plot depicting drugs characterized by a statistically significant correlation between area under concentration–response curve (AUC) response measurements and SU_LIVER enrichment scores. Green dots represent drugs that were more effective against well-differentiated cell lines compared to poorly differentiated ones, while red dots mark drugs that were relatively more effective against poorly differentiated HCC lines. Grey dots indicate drugs without a significant correlation between their effect and the differentiation status of cell lines. The horizontal dashed line marks the highest *p*-value corresponding to an adjusted *p*-value < 0.3, whereas the two vertical dashed lines mark Spearman’s ρ values equal to –0.5 and 0.5; x-axis: Spearman’s ρ, y-axis: *p*-value in negative log_10_ scale.

We next attempted to associate the drug-specific response of examined HCC lines with their differentiation status by correlating available drug-sensitivity AUC measurements with SU_LIVER enrichment scores. The volcano plot illustrated in Figure 10 demonstrates significant correlations (either positive or negative) between AUC measurements and SU_LIVER enrichment scores for 34 out of the 344 investigated drugs. Poorly differentiated cell lines were significantly more sensitive compared to well-differentiated ones against 11 investigated drugs, while, conversely, well-differentiated cell lines were relatively more sensitive against a panel of 23 investigated drugs, including but not limited to various tyrosine kinase inhibitors (TKIs). Table S8 provides a full listing of the correlation analysis results for all 344 studied drugs.

### 3.5. SU_LIVER-Based Clustering of HCC Patients Associated with the Assigned Tumor Grade

**Figure 11:**
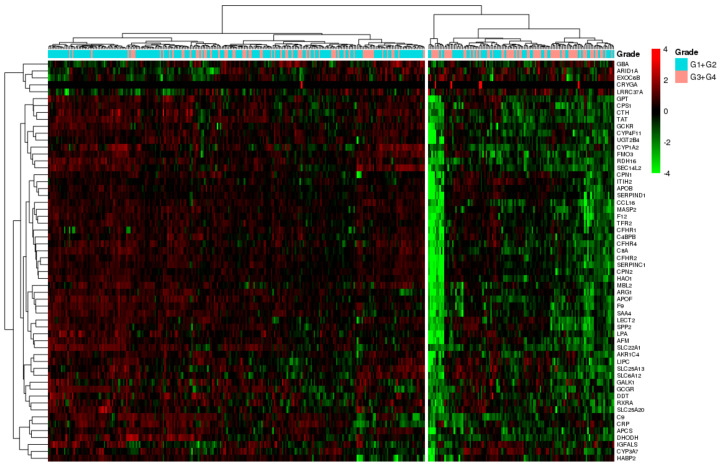
Heatmap illustrating the hierarchical clustering of HCC tumors (columns) based on the full set of SU_LIVER genes (rows). Scaled values indicate relative downregulation (green color) or upregulation (red color) of gene expression. HCC tumors are annotated by color according to their documented histological grade (G1+G2 versus G3+G4).

In order to explore the potential connection between acquired results from examined HCC lines and HCC patients’ data, we next tried to compare the differentiation characteristics of 354 tumors from the HCC TCGA cohort by hierarchical clustering based on the expression of SU_LIVER gene-set, as this was the main gene-set applied throughout the exploratory, ssGSEA, and drug screening analyses in HCC lines. Heatmap representation of the results (Figure 11) revealed two main obvious clusters. One major cluster consisted of 237 mostly well/moderately differentiated tumors (n_G1+G2_ = 172, n_G3+G4_ = 65) that generally overexpressed SU_LIVER genes, and a second smaller cluster contained 117 mostly poorly/undifferentiated tumors (n_G1+G2_ = 52, n_G3+G4_ = 65) that overall exhibited lower gene expression levels. Notably, as shown by the χ^2^- test, there was a statistically significant association (*p*-value = 2.41 × 10^-7^) between the formed clusters and the assigned tumor histological grading, a clear indicator of the degree of tumor differentiation.

## 4. Discussion

The use of current systems biology methodologies has greatly advanced the global investigation of intertumor heterogeneity in connection to drug-specific sensitivity/resistance, paving the way for the evolution of future therapeutic breakthroughs, including biomarker-driven treatments and precision medicine [6,22,36,37]. To this end, we applied an entirely computational approach implementing a multi-level bioinformatics analysis of publicly available transcriptomic, proteomic, and drug-screening datasets which enabled us to explore the molecular diversity of a large panel of established HCC lines and its association to drug responsiveness. 

Exploratory data analysis and a subsequent ssGSEA approach on DNA microarray data led to the classification of investigated cell lines into two main subgroups mainly defined by their respective differentiation status: a group of poorly and a group of well-differentiated cell lines overexpressing genes that are specifically upregulated in normal human liver tissues, and therefore were considered to retain a liver-like epithelial molecular profile. Notably, this subgrouping of HCC lines proved to be highly consistent at the proteome level and was further supported by hierarchical clustering based on an EMT gene signature. As EMT is a process highly implicated in tumor cell de-differentiation [29,38], this result provided complementary information to the differentiation profiling of HCC lines. It is recognized that high-grade poorly differentiated tumors are characterized by increased aggressiveness and poor prognosis [39]; consequently, histological grading of HCC presents an important prognostic marker along with various other molecular traits [40]. Although epithelial and/or mixed epithelial–mesenchymal characteristics are no longer considered to be completely disentangled from aggressive phenotypes [41], there is strong evidence that EMT is tightly associated with increased HCC growth and metastasis [42]. For this reason, several recent endeavors have generated and/or surveyed transcriptome and proteome data in various cancer cell line models —including HCC— in order to identify expression patterns connected to differentiation/EMT-related characteristics [6,22,36]; hence, the present work aspired to contribute accordingly. As a matter of fact, the differential gene and protein expression analysis conducted in order to explore the distinctive molecular background of poorly versus well-differentiated HCC lines highlighted 935 DEGs and 16 DEPs. Notably, the expression of almost all the genes encoding the identified DEPs was also accordingly modified, thus indicating that the observed changes in protein levels were attributed to a diverse transcriptional regulation of the corresponding genes. The most outstanding DEP findings included downregulation of the important epithelial/EMT marker ECADHERIN (*CDH1*) [43] and upregulation of CAVEOLIN1 (*CAV1*) [44] and PAI1 (*SERPINE1*) [45], both associated with increased aggressiveness, invasion, and metastasis. 

Subsequent functional enrichment analyses of identified DEGs against GO and Reactome Pathway databases were performed to efficiently elucidate significantly associated biological processes and pathways, while acquired enrichment data were further aggregated by *Bioinfominer* to identify core systemic biological processes/pathways and hub driver-gene signatures. Results from both ontological databases commonly revealed a specific pattern of differentially regulated processes and pathways involved in wound healing, hemostasis, coagulation, platelet dynamics, fibrinolysis, ECM remodeling, and cell migration/motility, as indicated by the high recurrence of relevant terminologies in the lists of the top 30 significantly enriched GO biological processes, and of 28 Reactome Pathway terms. Notably, the majority of these processes/pathways are biologically inter-connected and are either directly or indirectly associated with cancer progression, invasive/metastatic potential and overall aggressiveness. Tumors are often characterized as wounds that do not heal, constantly remodeling the stroma cells’ microenvironment and reorganizing ECM. This complex procedure involves provisional biological mechanisms that include inflammatory responses, clotting cascade activation, fibrin formation and fibrinolysis, angiogenesis, and enhanced vasculature [46,47]. Therefore, the attained deregulation of relevant processes/pathways in poorly compared to well-differentiated HCC lines is highly indicative of their augmented malignant capacity and offers useful information about the roles of implicated genes. Moreover, terms related to altered/impaired metabolism (especially of lipids, cholesterol/steroids, and lipoproteins)—a well-established cancer hallmark sustaining cancer cell growth and proliferation [48]—were evidently present in both functional enrichment lists, pointing out the significant role of metabolism-related genes and functions in defining the differential characteristics of HCC subtypes, in agreement with a recent metabolism-focused study on liver cancer [49]. *Bioinfominer* analysis proposed two distinct hub driver-gene signatures (each based on GO and Reactome Pathway results, respectively) involving molecular agents playing major roles in multiple underlying biological mechanisms. A number of genes, including several metabolism-related apolipoproteins and fibrinogens, commonly appeared in both signatures (*APOA1, APOA2, APOB, FGG, FGA, F2, APOE, NR1H3, GAS6, ACOX1*). Interestingly, *GAS6* upregulation emerged as a prominent factor in poorly differentiated HCC lines, implicated in a variety of systemic ontological terms. This finding, along with the observed overexpression of *AXL* and *SNAI2* (Slug), points to a key role for Gas6/Axl pathway, known to promote invasion and migration in HCC through Slug activation [50]. Moreover, AXL acts as a crucial regulator of cancer-related EMT [51]; particularly in HCC, the cooperation between Gas6/Axl and TGF-β signaling pathways appears to be crucial in differentiation, EMT, and the advancement of invasion [52]. Suitably, the TGF-β pathway was represented in the respective GO-derived hub gene signature by upregulated *TGFB2* gene, while other TGF-β signaling-associated genes were identified as DEGs as well, including overexpressed *TGFB1*. Both *TGFB1* and *TGFB2* are recognized as being heavily linked to EMT and tumor progression [38,53]. Additional noteworthy driver DEGs included in hub gene signatures were those known to be involved in fibrinolysis and platelet degranulation, such as *SERPINE1* (upregulated) and *A2M* (downregulated). *SERPINE1*, along with the identified (overexpressed) DEGs *PLAU* and *PLAUR*, is centrally implicated in cancer angiogenesis [54], while *A2M* possesses antitumorigenic properties [55]. Furthermore, *FGF2* along and its corresponding receptor *FGFR1* (both upregulated) were also found in the identified hub driver-gene signatures. It is well established that FGF2/FGFR signaling deregulation is associated with aggressive tumor phenotypes and drug resistance [56], features recurrently linked to the poorly differentiated HCC subtypes. Additionally, *HNF4A*’s (downregulated) characterization as a distinctive molecular player between differentiation states of examined HCC lines was in fact anticipated, due to the pivotal role of this transcription factor in liver function and hepatocyte differentiation [57]. Finally, the ontological term “biological oxidations”—widely associated with oxidative stress and fatty acid metabolism in HCC [58]—was also demonstrated as systemic core process in the Reactome Pathway data, represented by *GNG11*, *GNG12*, *ACOX1*, and multiple apolipoprotein genes. 

Complementary information regarding potentially implicated biological mechanisms was subsequently gathered by performing supplementary gene-set enrichment analysis against curated datasets from MSigDB based on differential expression analysis data. Results once again highlighted a more aggressive phenotype for poorly differentiated HCC lines. It is worth noting that this group of HCC lines shared common upregulated genes with the S1 HCC subtype identified by Hoshida et al. in primary HCC—characterized by mesenchymal characteristics/active TGF-β signaling—while the group of better-differentiated ones resembled subtypes S2 and S3, retaining a more hepatocyte-like phenotype while overexpressing certain hepatoblast markers like *AFP* and *EPCAM* [35].

Lastly, the association of HCC differentiation status with drug sensitivity/resistance—explored by correlating cell line SU_LIVER enrichment scores with drug efficacy measurements for a panel of compounds—resulted in the identification of drugs that were more effective against poorly than well-differentiated cell lines, and vice versa. HCC lines in the well-differentiated group proved to be more sensitive than their counterparts against a larger number of investigated drugs/agents, including several TKIs such as those targeting EGFR (erlotinib), IGF1R (linsitinib, BMS-536924, BMS-754807), or other kinases (linifanib, masitinib, imatinib). EMT has been identified as a major contributor of acquired resistance against EGFR-TKIs in non-small-cell lung cancers [59], while protein-level pan-cancer studies have highlighted an EMT-status-dependent efficacy of several EGFR inhibitors and other targeted therapies [37]. Interestingly, as both genes were overexpressed in poorly differentiated cell lines, FGF2/FGFR1 activation in non-small-cell lung cancer has been proposed as an important EGFR-TKI-resistance-acquisition mechanism [60]. Furthermore, the present results for IGF1R and MDM2 inhibitors corroborated those of a recent study on numerous cancer liver cell lines providing experimental evidence of an augmented efficacy of IGF1R inhibitor linsitinib as well as of MDM2 inhibitor nutlin-3 against liver cancer lines with prominent hepatoblast/hepatocyte-like characteristics [36], thus supporting the validity of our approach. Additionally, the natural compound epigallocatechin-3-monogallate was identified as being comparatively more effective against well-differentiated HCC lines, in full agreement with previous studies highlighting HEP3B and HEPG2 cell lines as particularly responsive against that nutraceutical [61,62]. 

On the other hand, a smaller portion of examined drugs displayed an enhanced efficacy against the poorly differentiated group in comparison to well-differentiated HCC lines. Among them, it is worth discussing the role of three compounds, namely the retinoic acid receptor β (RARB) agonist AC55649, the SPHK1 inhibitor SKI-II, and the PKM2 activator ML203. All-trans retinoic acid (ATRA), a known pan-retinoic receptor agonist, has been found to regulate EMT and inhibit migration in breast cancer via the TGF-β pathway [63], a notion that might support the use of RARB agonists against poorly differentiated HCC with TGF-β-dependent EMT features. As for SPHK1, it is known to induce EMT in hepatoma cells through the promotion of *CDH1*/ECADHERIN degradation [64], and thus, SPHK1-inhibitors could be a reasonable option against mesenchymal-like HCC. Finally, PKM2 activation through ML203 presents an interesting prospect, since PKM2—a metabolic enzyme in glycolysis—is attracting growing attention due to a manifold possible involvement in cancer progression [65]. It has been shown that PKM2 activity is negatively regulated by the increased presence of CD44 (a known cancer biomarker), and this effect mediates the aggressive glycolytic phenotype of colon cancer cells [66]. Notably, the *CD44* gene was one of the most significantly upregulated DEGs in poorly differentiated HCC lines. It is thus possible that the compound ML203 could counteract the hampered PKM2 activation by overexpressed *CD44*, and therefore inhibit the glycolytic phenotype of poorly differentiated HCC. 

Although our analysis was entirely based on data from HCC lines, the acquired information of drug sensitivity in poorly and well-differentiated cell lines might be of clinical relevance for HCC patients with characterized tumor differentiation profiles. To this end, tumors from 354 patients from the HCC TCGA cohort were stratified on the basis of SU_LIVER gene-set expression data into two clusters statistically significant for tumor grade, one with samples generally displaying high/moderate differentiation and overexpression of SU_LIVER genes, and one with overall poorly/undifferentiated tumors and lower SU_LIVER gene expression levels. Since HCC patient clustering corroborated with the corresponding stratification of HCC lines into poorly and well-differentiated groups, the present TCGA investigation (a) broadened the reliability of SU_LIVER genes-based clustering as an informative analysis that significantly correlates gene expression pattern with the differentiation status of HCC lines and—very importantly—of patient tumors as well, and (b) in conjunction with the aforementioned drug-sensitivity data for HCC lines (also based on SU_LIVER enrichment scores), may offer preliminary clues in future clinical research for predicting drug efficiency in HCC patients possessing analogous gene expression characteristics and histological grade. However, it should be noted that cell-line models—lacking crucial interactions with immune/stromal cells and surrounding ECM—are not perfect representations of in vivo tumors and thus, cannot fully recapitulate the wide spectrum of tumor heterogeneity and consequently their response to drugs.

Certainly, the availability and the constantly growing volume of several types of -omics and drug-screening data in multiple public resources make their computational integration and investigation of their biological/clinical relevance a real challenge in ongoing cancer research. Focusing on HCC—in addition to the currently presented analysis of transcriptomic and proteomic data—future studies exploiting available genomics data, including DNA alterations, especially in genes and gene-expression-regulatory elements, as well as evaluating metabolome variations, are required in order to provide further insights into the mechanisms underlying HCC heterogeneity. Preliminary bioinformatics analysis by our group, which was based on accessible data for coding-gene mutations and copy-number variations (deletions/amplifications) in HCC lines provided some interesting initial observations that merit further investigation. 

## 5. Conclusions

The present work, by using a thorough in silico analysis of publicly available transcriptomic, proteomic, and drug-screening data, classified a large panel of HCC lines into two representative differentiation subtypes of either higher or lower differentiation status, each exhibiting a discrete sensitivity pattern against numerous evaluated drugs. Furthermore, the identification of two hub driver DEGs signatures across informative ontology/pathway databases provided evidence on functionally significant biomarkers, thus offering a starting basis for mechanistic and pharmacogenomic studies. Overall, the described methodologies provide a comprehensive cost-effective computational framework, able to be applied in any model cancer cell lines as long as relevant -omics and drug-screening data are accessible in dedicated repositories, which allows the investigation of inherent tumor molecular diversity and the design of therapeutic regimes effective for each cancer subtype.

## Figures and Tables

**Table 1:** HCC lines with available gene and protein/phosphoprotein expression data used in data analysis.

Cell Line Name	Cancer Type	Cell Line Name	Cancer Type	Cell Line Name	Cancer Type
HEP3B217	HCC	JHH4	HCC	SNU387	HCC
HEPG2	HCC	JHH5	HCC	SNU423	HCC
HLF	HCC	JHH6	HCC	SNU449	HCC
HUH1	HCC	JHH7	HCC	SNU475	HCC
HUH7	HCC	LI7	HCC	SNU761	HCC
JHH1	HCC	PLCPRF5	HCC	SNU878	HCC
JHH2	HCC	SKHEP1	Adenocarcinoma	SNU886	HCC

Blue font indicates cell lines with drug sensitivity data available from the Cancer Therapeutics Response Portal (CTRPv2) and used in pharmacogenomic analysis. HCC: Hepatocellular carcinoma

**Table 2:** Bioinfominer gene signature (poorly versus well-differentiated cell lines) based on implicated GO systemic processes. The signature consisted of 41 hub driver-genes, which are presented along with their corresponding number of implicated systemic processes and log_2_(fold-changes).

Gene Symbol	Gene Name	Systemic Processes	log_2_ (Fold-Change)
*APOA1*	*apolipoprotein A1*	19	–5.81
*APOE*	*apolipoprotein E*	14	–4.11
*APOA2*	*apolipoprotein A2*	12	–8.62
*CAV1*	*caveolin 1*	12	3.67
*SERPINF2*	*serpin family F member 2*	12	–2.43
*TGFB2*	*transforming growth factor beta 2*	11	2.84
*AGTR1*	*angiotensin II receptor type 1*	11	–2.95
*ANXA1*	*annexin A1*	11	3.14
*AGT*	*angiotensinogen*	10	–5.06
*APOH*	*apolipoprotein H*	10	–7.09
*FGF2*	*fibroblast growth factor 2*	10	2.84
*SCARB1*	*scavenger receptor class B member 1*	10	–1.57
*APOC3*	*apolipoprotein C3*	10	–4.89
*APOC1*	*apolipoprotein C1*	10	–5.47
*THBS1*	*thrombospondin 1*	10	2.21
*APOB*	*apolipoprotein B*	9	–6.71
*NRP1*	*neuropilin 1*	9	1.80
*FGG*	*fibrinogen gamma chain*	9	–6.37
*FGA*	*fibrinogen alpha chain*	9	–5.49
*FGB*	*fibrinogen beta chain*	9	–4.93
*SERPINE1*	*serpin family E member 1*	9	2.98
*CEACAM1*	*carcinoembryonic antigen related cell adhesion molecule 1*	8	–2.67
*DYSF*	*dysferlin*	8	1.76
*NR1H4*	*nuclear receptor subfamily 1 group H member 4*	8	–4.08
*TSPO*	*translocator protein*	8	2.48
*CPB2*	*carboxypeptidase B2*	8	–5.98
*HNF4A*	*hepatocyte nuclear factor 4 alpha*	8	–1.24
*XBP1*	*X-box binding protein 1*	8	–1.38
*ANGPTL3*	*angiopoietin like 3*	7	–3.90
*NR1H3*	*nuclear receptor subfamily 1 group H member 3*	7	–1.42
*FLNA*	*filamin A*	7	2.32
*F2*	*coagulation factor II, thrombin*	7	–5.12
*BAD*	*BCL2 associated agonist of cell death*	7	0.96
*LIPC*	*lipase C, hepatic type*	7	–4.24
*GAS6*	*growth arrest specific 6*	7	2.39
*VTN*	*vitronectin*	7	–5.08
*FGFR1*	*fibroblast growth factor receptor 1*	7	1.49
*ARG1*	*arginase 1*	7	–2.79
*CYBA*	*cytochrome b-245 alpha chain*	7	–3.33
*SULT1E1*	*sulfotransferase family 1E member 1*	4	–2.07
*ACOX1*	*acyl-CoA oxidase 1*	4	–1.10

**Table 3:** Bioinfominer gene signature (poorly versus well-differentiated cell lines) based on implicated Reactome Pathway systemic processes. The signature consisted of 21 hub driver-genes, which are presented along with their corresponding number of implicated systemic processes and log_2_ (fold-changes).

Gene Symbol	Gene Name	Systemic Processes	log_2_(Fold- Change)
*APOA1*	*apolipoprotein A1*	5	–5.81
*APOA2*	*apolipoprotein A2*	4	–8.62
*APOB*	*apolipoprotein B*	4	–6.71
*ALB*	*albumin*	4	–8.42
*SERPINC1*	*serpin family C member 1*	3	–2.66
*GNG11*	*G protein subunit gamma 11*	3	3.24
*GNG12*	*G protein subunit gamma 12*	3	2.41
*FGG*	*fibrinogen gamma chain*	3	–6.37
*FGA*	*fibrinogen alpha chain*	3	–5.49
*F2*	*coagulation factor II, thrombin*	3	–5.12
*KNG1*	*kininogen 1*	3	–1.82
*APOE*	*apolipoprotein E*	3	–4.11
*NR1H3*	*nuclear receptor subfamily 1 group H member 3*	3	–1.42
*GAS6*	*growth arrest specific 6*	3	2.39
*PROC*	*protein C, inactivator of coagulation factors Va and VIIIa*	3	–3.25
*A2M*	*alpha-2-macroglobulin*	3	–5.26
*SERPIND1*	*serpin family D member 1*	3	–5.60
*F5*	*coagulation factor V*	3	–4.30
*ACOX1*	*acyl-CoA oxidase 1*	3	–1.10
*PRKACB*	*protein kinase cAMP-activated catalytic subunit beta*	3	1.11
*TF*	*transferrin*	3	–8.73

## Supplementary Materials

The following are available online at https://www.mdpi.com/2073-4425/11/6/623/s1, Figure S1: Correlation matrix for HCC line pairs based on the expression of the 500 genes with the largest cross-sample variation, Figure S2: Correlation matrix for HCC line pairs based on the expression of 214 proteins/phosphoproteins, Figure S3: BIC results for optimal number of *k*-means clusters based on PC1 scores, Figure S4: PCA of HCC lines based on CCLE RNA-seq gene expression data, Figure S5: BIC results for optimal number of *k*-means clusters based on computed cell-line ssGSEA SU_LIVER enrichment scores, Figure S6: Heatmap illustrating the scaled expression values of HCC lines regarding identified DEGs, Figure S7: Heatmap illustrating the scaled expression values of HCC lines regarding identified DEPs, Figure S8: GO systemic processes and involved hub genes, Figure S9: Reactome Pathway systemic processes and involved hub genes, Table S1: ssGSEA scores based on PC1 loadings, Table S2: ssGSEA scores for individual cell lines, Table S3: List of DEGs, Table S4: List of DEPs, Table S5: GO enrichment results, Table S6: Reactome Pathway enrichment results, Table S7: Gene-set enrichment results from *camera*, Table S8: Drug-response correlation with SU_LIVER enrichment score results.

## References

[1] Forner A., Reig M., Bruix J. (2018). Hepatocellular carcinoma. Lancet.

[2] Fujiwara N., Friedman S.L., Goossens N., Hoshida Y. (2018). Risk factors and prevention of hepatocellular carcinoma in the era of precision medicine. J. Hepatol..

[3] Aravalli R.N., Cressman E.N.K., Steer C.J. (2013). Cellular and molecular mechanisms of hepatocellular carcinoma: An update. Arch. Toxicol..

[4] Llovet J.M., Montal R., Sia D., Finn R.S. (2018). Molecular therapies and precision medicine for hepatocellular carcinoma. Nat. Rev. Clin. Oncol..

[5] Katt M.E., Placone A.L., Wong A.D., Xu Z.S., Searson P.C. (2016). In vitro tumor models: Advantages, disadvantages, variables, and selecting the right platform. Front. Bioeng. Biotechnol..

[6] Berg K.C.G., Eide P.W., Eilertsen I.A., Johannessen B., Bruun J., Danielsen S.A., Bjørnslett M., Meza-Zepeda L.A., Eknæs M., Lind G.E. (2017). Multi-omics of 34 colorectal cancer cell lines—A resource for biomedical studies. Mol. Cancer.

[7] Barretina J., Caponigro G., Stransky N., Venkatesan K., Margolin A.A., Kim S., Wilson C.J., Lehár J., Gregory V., Sonkin D. (2012). The Cancer Cell Line Encyclopedia enables predictive modeling of anticancer drug sensitivity. Nature.

[8] Ghandi M., Huang F.W., Jané-Valbuena J., Kryukov G.V., Lo C.C., McDonald E.R., Barretina J., Gelfand E.T., Bielski C.M., Li H. (2019). Next-generation characterization of the Cancer Cell Line Encyclopedia. Nature.

[9] Park J., Lee J., Kang M., Park K., Jeon Y., Lee H., Kwon H., Park H., Yeo K., Lee K. (1995). Characterization of cell lines established from human hepatocellular carcinoma. Int. J. Cancer.

[10] Heffelfinger S.C., Hawkins H.H., Barrish J., Taylor L., Darlington G.J. (1992). SK HEP-1: A human cell line of endothelial origin. Cell. Dev. Biol. Anim..

[11] Rebouissou S., Zucman-Rossi J., Moreau R., Qiu Z., Hui L. (2017). Note of caution: Contaminations of hepatocellular cell lines. J. Hepatol..

[12] Seashore-Ludlow B., Rees M.G., Cheah J.H., Coko M., Price E.V., Coletti M.E., Jones V., Bodycombe N.E., Soule C.K., Gould J. (2015). Harnessing connectivity in a large-scale small-molecule sensitivity dataset. Cancer Discov..

[13] Basu A., Bodycombe N.E., Cheah J.H., Price E.V., Liu K., Schaefer G.I., Ebright R.Y., Stewart M.L., Ito D., Wang S. (2013). An interactive resource to identify cancer genetic and lineage dependencies targeted by small molecules. Cell.

[14] Ally A., Balasundaram M., Carlsen R., Chuah E., Clarke A., Dhalla N., Holt R.A., Jones S.J.M., Lee D., Ma Y. (2017). Comprehensive and integrative genomic characterization of hepatocellular carcinoma. Cell.

[15] Li B., Dewey C.N. (2011). RSEM: Accurate transcript quantification from RNA-Seq data with or without a reference genome. BMC Bioinform..

[16] Robinson M.D., Oshlack A. (2010). A scaling normalization method for differential expression analysis of RNA-seq data. Genome Biol..

[17] Rohart F., Gautier B., Singh A., Lê Cao K.A. (2017). mixOmics: An R package for ‘omics feature selection and multiple data integration. PLoS Comput. Biol..

[18] Wang H., Song M. (2011). Ckmeans.1d.dp: Optimal k-means clustering in one dimension by dynamic programming. R Journal.

[19] Schwarz G. (1978). Estimating the dimension of a model. Ann. Stat..

[20] Hänzelmann S., Castelo R., Guinney J. (2013). GSVA: Gene set variation analysis for microarray and RNA-Seq data. BMC Bioinform..

[21] Barbie D.A., Tamayo P., Boehm J.S., Kim S.Y., Moody S.E., Dunn I.F., Schinzel A.C., Sandy P., Meylan E., Scholl C. (2009). Systematic RNA interference reveals that oncogenic KRAS-driven cancers require TBK1. Nature.

[22] Koplev S., Lin K., Dohlman A.B., Ma’ayan A. (2018). Integration of pan-cancer transcriptomics with RPPA proteomics reveals mechanisms of epithelial-mesenchymal transition. PLoS Comput. Biol..

[23] Ritchie M.E., Phipson B., Wu D., Hu Y., Law C.W., Shi W., Smyth G.K. (2015). *Limma* powers differential expression analyses for RNA-sequencing and microarray studies. Nucleic Acids Res..

[24] Mccarthy D.J., Smyth G.K. (2009). Testing significance relative to a fold-change threshold is a TREAT. Bioinformatics.

[25] Blighe K. Publication-ready volcano plots with enhanced colouring and labeling. https://github.com/kevinblighe/EnhancedVolcano.

[26] Koutsandreas T., Binenbaum I., Pilalis E., Valavanis I., Papadodima O., Chatziioannou A. (2016). Analyzing and visualizing genomic complexity for the derivation of the emergent molecular networks. Int. J. Monit. Surveill. Technol. Res..

[27] Lhomond S., Avril T., Dejeans N., Voutetakis K., Doultsinos D., McMahon M., Pineau R., Obacz J., Papadodima O., Jouan F. (2018). Dual IRE1 RNase functions dictate glioblastoma development. EMBO Mol. Med..

[28] Wu D., Smyth G.K. (2012). Camera: A competitive gene set test accounting for inter-gene correlation. Nucleic Acids Res..

[29] Wang H., Unternaehrer J.J. (2019). Epithelial-mesenchymal transition and cancer stem cells: At the crossroads of differentiation and dedifferentiation. Dev. Dyn..

[30] Su A.I., Cooke M.P., Ching K.A., Hakak Y., Walker J.R., Wiltshire T., Orth A.P., Vega R.G., Sapinoso L.M., Moqrich A. (2002). Large-scale analysis of the human and mouse transcriptomes. Proc. Natl. Acad. Sci. USA.

[31] Hsiao L.L., Dangond F., Yoshida T., Hong R., Jensen R.V., Misra J., Dillon W., Lee K.F., Clark K.E., Haverty P. (2002). A compendium of gene expression in normal human tissues. Physiol. Genom..

[32] Anastassiou D., Rumjantseva V., Cheng W., Huang J., Canoll P.D., Yamashiro D.J., Kandel J.J. (2011). Human cancer cells express Slug-based epithelial-mesenchymal transition gene expression signature obtained in vivo. BMC Cancer.

[33] Elvidge G.P., Glenny L., Appelhoff R.J., Ratcliffe P.J., Ragoussis J., Gleadle J.M. (2006). Concordant regulation of gene expression by hypoxia and 2-oxoglutarate-dependent dioxygenase inhibition: The role of HIF-1α, HIF-2α, and other pathways. J. Biol. Chem..

[34] Wu Y., Siadaty M.S., Berens M.E., Hampton G.M., Theodorescu D. (2008). Overlapping gene expression profiles of cell migration and tumor invasion in human bladder cancer identify metallothionein 1E and nicotinamide N-methyltransferase as novel regulators of cell migration. Oncogene.

[35] Hoshida Y., Nijman S.M.B., Kobayashi M., Chan J.A., Brunet J.P., Chiang D.Y., Villanueva A., Newell P., Ikeda K., Hashimoto M. (2009). Integrative transcriptome analysis reveals common molecular subclasses of human hepatocellular carcinoma. Cancer Res..

[36] Caruso S., Calatayud A.L., Pilet J., La Bella T., Rekik S., Imbeaud S., Letouzé E., Meunier L., Bayard Q., Rohr-Udilova N. (2019). Analysis of liver cancer cell lines identifies agents with likely efficacy against hepatocellular carcinoma and markers of response. Gastroenterology.

[37] Li J., Zhao W., Akbani R., Liu W., Ju Z., Ling S., Vellano C.P., Roebuck P., Yu Q., Eterovic A.K. (2017). Characterization of human cancer cell lines by reverse-phase protein arrays. Cancer Cell.

[38] Nieto M.A., Huang R.Y.-J., Jackson R.A., Thiery J.P. (2016). EMT: 2016. Cell.

[39] Dongre A., Weinberg R.A. (2019). New insights into the mechanisms of epithelial–mesenchymal transition and implications for cancer. Nat. Rev. Mol. Cell Biol..

[40] Martins-Filho S.N., Paiva C., Azevedo R.S., Alves V.A.F. (2017). Histological grading of hepatocellular carcinoma-a systematic review of literature. Front. Med..

[41] Williams E.D., Gao D., Redfern A., Thompson E.W. (2019). Controversies around epithelial–mesenchymal plasticity in cancer metastasis. Nat. Rev. Cancer.

[42] Giannelli G., Koudelkova P., Dituri F., Mikulits W. (2016). Role of epithelial to mesenchymal transition in hepatocellular carcinoma. J. Hepatol..

[43] Schmalhofer O., Brabletz S., Brabletz T. (2009). E-cadherin, β-catenin, and ZEB1 in malignant progression of cancer. Cancer Metastasis Rev..

[44] Ting Tse E.Y., Fat Ko F.C., Kwan Tung E.K., Chan L.K., Wah Lee T.K., Wai Ngan E.S., Man K., Tsai Wong A.S., Ng I.O.L., Ping Yam J.W. (2012). Caveolin-1 overexpression is associated with hepatocellular carcinoma tumourigenesis and metastasis. J. Pathol..

[45] Li S., Wei X., He J., Tian X., Yuan S., Sun L. (2018). Plasminogen activator inhibitor-1 in cancer research. Biomed. Pharmacother..

[46] Dvorak H.F. (2015). Tumors: Wounds that do not heal-redux. Cancer Immunol. Res..

[47] Foster D.S., Jones R.E., Ransom R.C., Longaker M.T., Norton J.A. (2018). The evolving relationship of wound healing and tumor stroma. JCI Insight.

[48] Hanahan D., Weinberg R.A. (2011). Hallmarks of cancer: The next generation. Cell.

[49] Nwosu Z.C., Battello N., Rothley M., Piorońska W., Sitek B., Ebert M.P., Hofmann U., Sleeman J., Wölfl S., Meyer C. (2018). Liver cancer cell lines distinctly mimic the metabolic gene expression pattern of the corresponding human tumours. J. Exp. Clin. Cancer Res..

[50] Lee H.J., Jeng Y.M., Chen Y.L., Chung L., Yuan R.H. (2014). Gas6/Axl pathway promotes tumor invasion through the transcriptional activation of slug in hepatocellular carcinoma. Carcinogenesis.

[51] Antony J., Huang R.Y.J. (2017). AXL-driven EMT state as a targetable conduit in cancer. Cancer Res..

[52] Reichl P., Dengler M., van Zijl F., Huber H., Führlinger G., Reichel C., Sieghart W., Peck-Radosavljevic M., Grubinger M., Mikulits W. (2015). Axl activates autocrine transforming growth factor-β signaling in hepatocellular carcinoma. Hepatology.

[53] Ikushima H., Miyazono K. (2010). TGFΒ 2 signalling: A complex web in cancer progression. Nat. Rev. Cancer.

[54] Kwaan H.C., Lindholm P.F. (2019). Fibrin and fibrinolysis in cancer. Semin. Thromb. Hemost..

[55] Kurz S., Thieme R., Amberg R., Groth M., Jahnke H.G., Pieroh P., Horn L.C., Kolb M., Huse K., Platzer M. (2017). The anti-tumorigenic activity of A2M—A lesson from the naked mole-rat. PLoS ONE.

[56] Akl M.R., Nagpal P., Ayoub N.M., Tai B., Prabhu S.A., Capac C.M., Gliksman M., Goy A., Suh K.S. (2016). Molecular and clinical significance of fibroblast growth factor 2 (FGF2/bFGF) in malignancies of solid and hematological cancers for personalized therapies. Oncotarget.

[57] Ning B.F., Ding J., Yin C., Zhong W., Wu K., Zeng X., Yang W., Chen Y.X., Zhang J.P., Zhang X. (2010). Hepatocyte nuclear factor 4α suppresses the development of hepatocellular carcinoma. Cancer Res..

[58] Wang M., Han J., Xing H., Zhang H., Li Z., Liang L., Li C., Dai S., Wu M., Shen F. (2016). Dysregulated fatty acid metabolism in hepatocellular carcinoma. Hepatic Oncol..

[59] Zhu X., Chen L., Liu L., Niu X. (2019). EMT-Mediated Acquired EGFR-TKI Resistance in NSCLC: Mechanisms and Strategies. Front. Oncol..

[60] Terai H., Soejima K., Yasuda H., Nakayama S., Hamamoto J., Arai D. (2013). Activation of the FGF2-FGFR1 autocrine pathway: A novel mechanism of acquired resistance to gefitinib in NSCLC. Mol. Cancer Res..

[61] Michailidou M., Melas I., Messinis D., Klamt S., Alexopoulos L., Kolisis F., Loutrari H. (2015). Network-based analysis of nutraceuticals in human hepatocellular carcinomas reveals mechanisms of chemopreventive action. CPT Pharmacomet. Syst. Pharmacol..

[62] Agioutantis P.C., Kotsikoris V., Kolisis F.N., Loutrari H. (2020). RNA-seq data analysis of stimulated hepatocellular carcinoma cells treated with epigallocatechin gallate and fisetin reveals target genes and action mechanisms. Comput. Struct. Biotechnol. J..

[63] Zanetti A., Affatato R., Centritto F., Fratelli M., Kurosaki M., Barzago M.M., Bolis M., Terao M., Garattini E., Paroni G. (2015). All-trans-retinoic acid modulates the plasticity and inhibits the motility of breast cancer cells role of notch1 and transforming growth factor (TGF β). J. Biol. Chem..

[64] Liu H., Ma Y., He H.W., Zhao W.L., Shao R.G. (2017). SPHK1 (sphingosine kinase 1) induces epithelial-mesenchymal transition by promoting the autophagy-linked lysosomal degradation of CDH1/E-cadherin in hepatoma cells. Autophagy.

[65] Hsu M.C., Hung W.C. (2018). Pyruvate kinase M2 fuels multiple aspects of cancer cells: From cellular metabolism, transcriptional regulation to extracellular signaling. Mol. Cancer.

[66] Tamada M., Nagano O., Tateyama S., Ohmura M., Yae T., Ishimoto T., Sugihara E., Onishi N., Yamamoto T., Yanagawa H. (2012). Modulation of glucose metabolism by CD44 contributes to antioxidant status and drug resistance in cancer cells. Cancer Res..

