# Supplementary material for: Computational Analysis of Transcriptomic and Proteomic Data for Deciphering Molecular Heterogeneity and Drug Responsiveness in Model Human Hepatocellular Carcinoma Cell Lines
Source: Genes (Basel). 2020 Jun 5;11(6):623. doi: 10.3390/genes11060623 (PMC7349788; doi:10.3390/genes11060623)
Supplement: Supplementary file 1 [file genes-11-00623-s001.zip › Supplementary Figure Captions.docx]

**Figure S1.** Correlation matrix for HCC lines pairs based on the expression of 500 genes with the largest cross-sample variation. Red and blue color indicates negative or positive Pearson’s correlation, respectively.

**Figure S2.** Correlation matrix for HCC lines pairs based on the expression of all available 214 proteins/phosphoproteins in the RPPA dataset. Red and blue color indicates negative or positive Pearson’s correlation, respectively.

**Figure S3.** BIC results for optimal number of *k*-means clusters based on PC1 scores.

**Figure S4.** PCA of HCC lines based on CCLE RNAseq gene expression data **(A)** for 488 common genes out of the 500 most variant genes in the microarray dataset **(B)** for the 500 most variant genes in the RNAseq dataset. 21 HCC lines are indicated by blue color. Almost identical (A) or extremely similar (B) results with Figure 1A can be observed. RNAseq data were downloaded as raw gene-level counts (file name CCLE_RNAseq_genes_counts_20180929.gct.gz) from CCLE, consequently normalized by TMM method of normalization implemented in the edgeR package and finally transformed in log_2_ scale.

**Figure S5.** BIC results for optimal number of *k*-means clusters based on computed cell line ssGSEA SU_LIVER enrichment scores.

**Figure S6.** Heatmap illustrating the gene expression patterns of HCC lines (columns) based on the identified DEGs (rows). Scaled values indicate relative downregulation (green color) or upregulation (red color) of gene expression.

**Figure S7.** Heatmap illustrating the protein expression patterns of HCC lines (columns) based on the identified DEPs (rows). Scaled values indicate relative downregulation (green color) or upregulation (red color) of protein expression.

**Figure S8.** GO systemic processes and involved hub genes. Red color indicates overexpressed and blue color underexpressed genes.

**Figure S9.** Reactome Pathway systemic processes and involved hub genes. Red color indicates overexpressed and blue color underexpressed genes.
