# Supplementary figures and images for: Computational Analysis of Transcriptomic and Proteomic Data for Deciphering Molecular Heterogeneity and Drug Responsiveness in Model Human Hepatocellular Carcinoma Cell Lines
Source: Genes (Basel). 2020 Jun 5;11(6):623. doi: 10.3390/genes11060623 (PMC7349788; doi:10.3390/genes11060623)

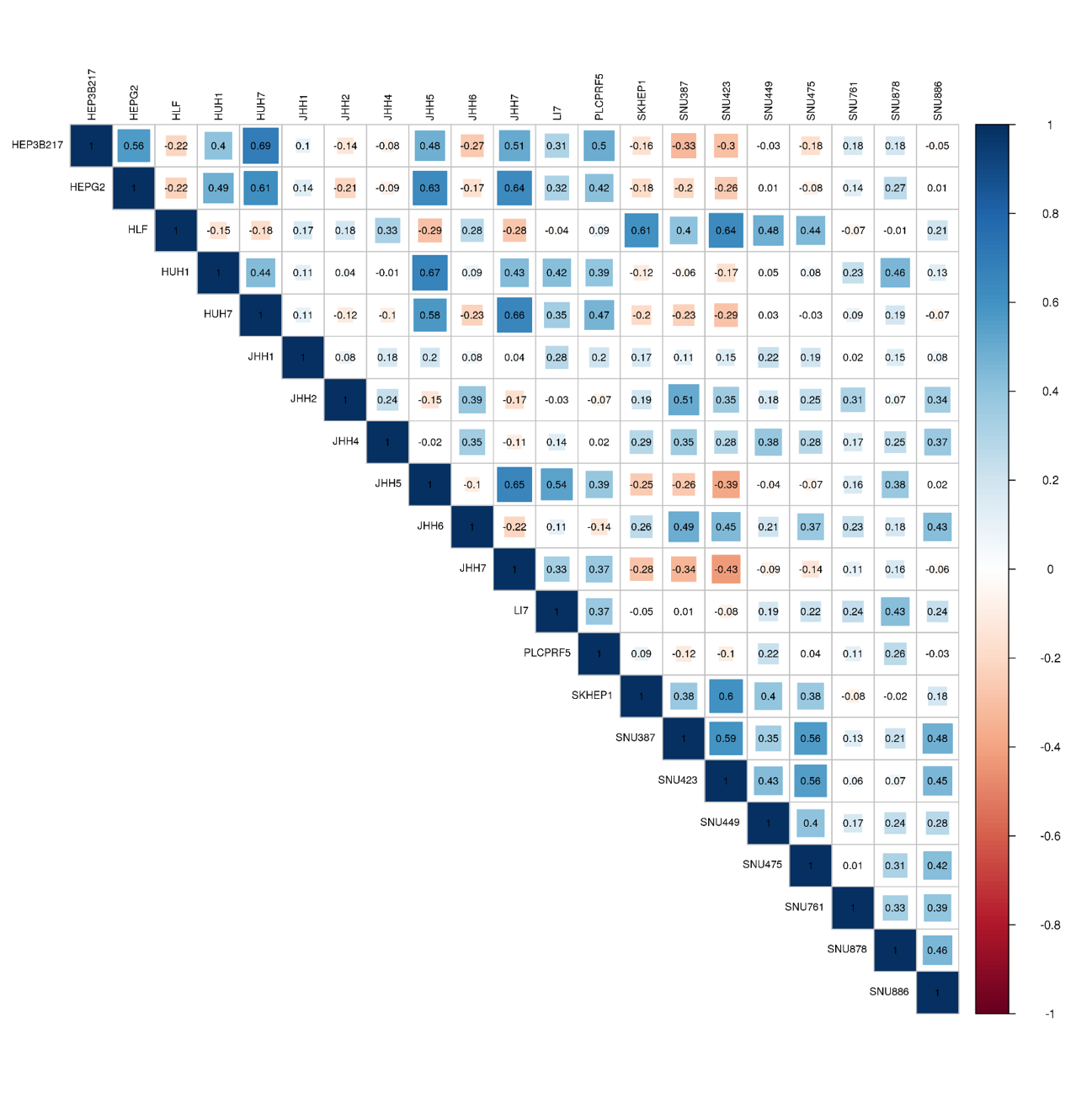

Supplement: Supplementary file 1 [file genes-11-00623-s001.zip › Figure S1.tif]

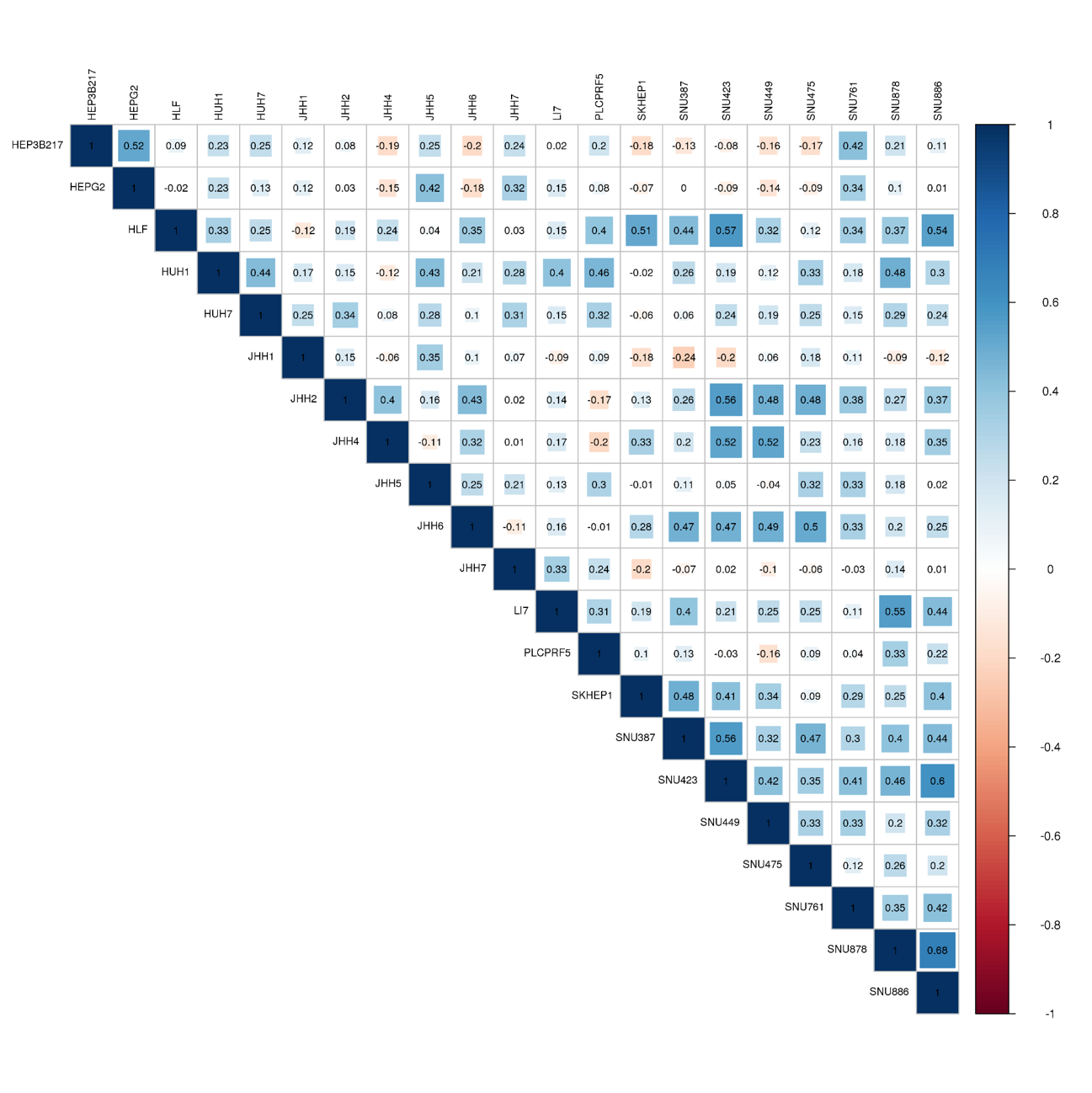

Supplement: Supplementary file 1 [file genes-11-00623-s001.zip › Figure S2.tif]

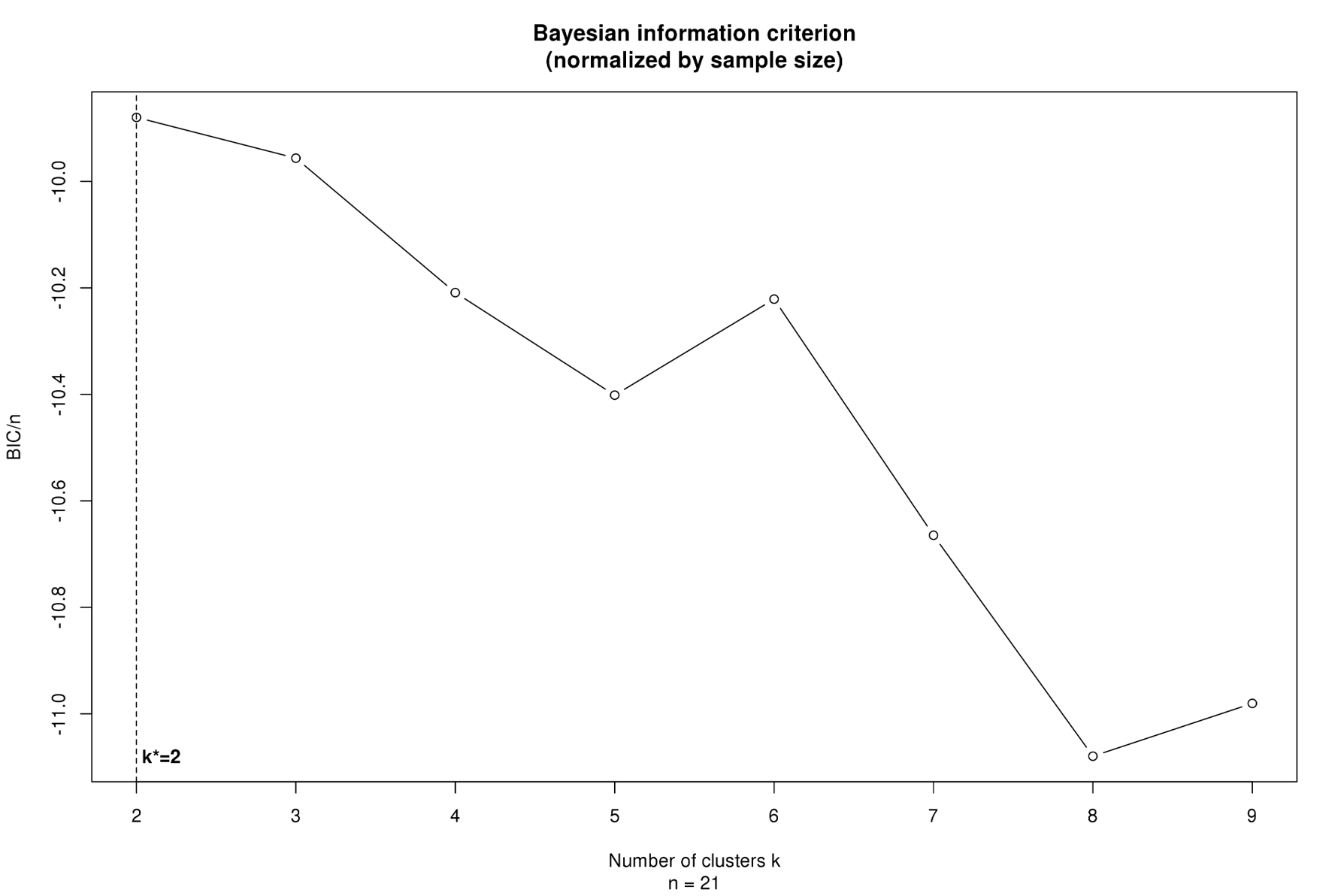

Supplement: Supplementary file 1 [file genes-11-00623-s001.zip › Figure S3.tif]

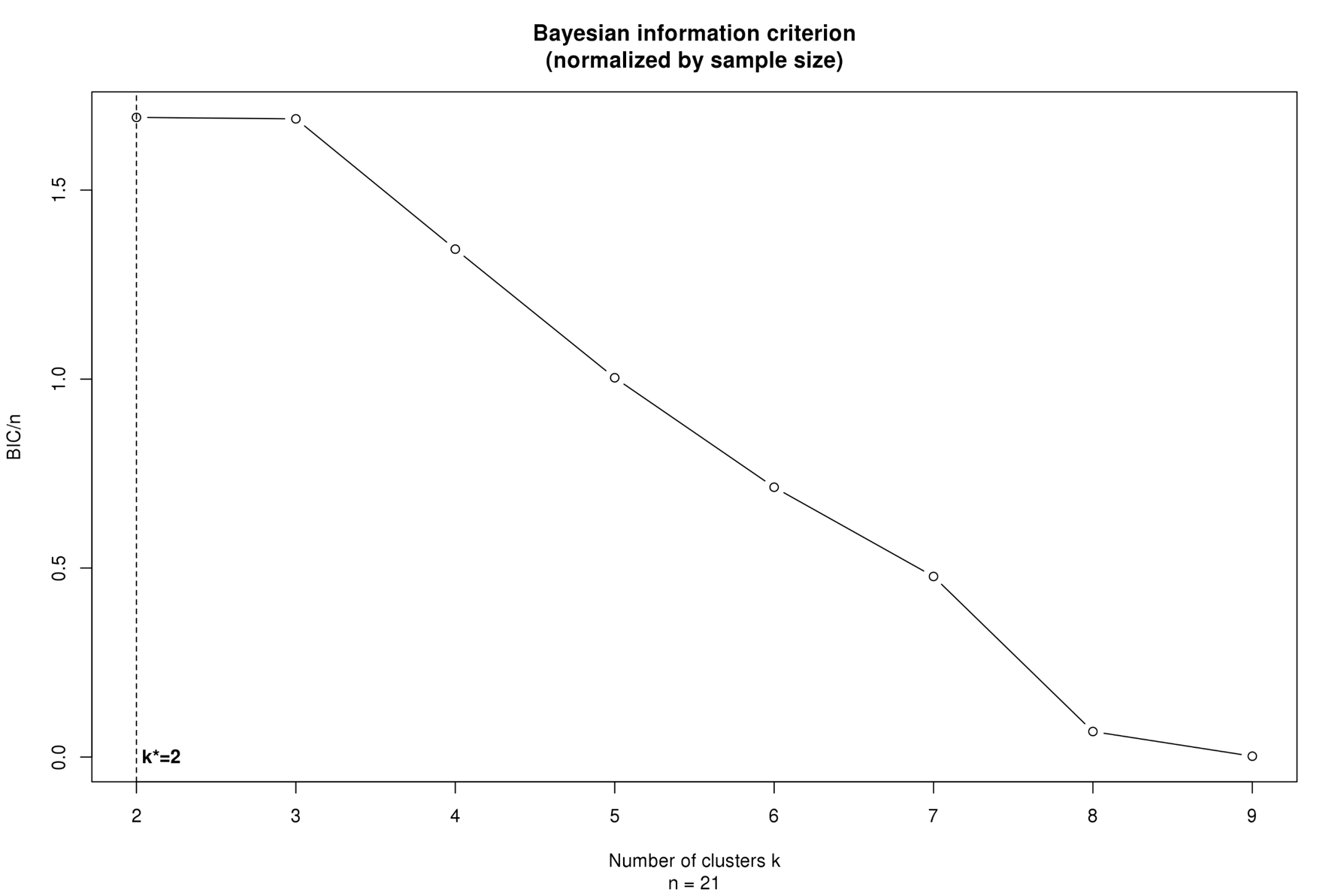

Supplement: Supplementary file 1 [file genes-11-00623-s001.zip › Figure S5.tif]

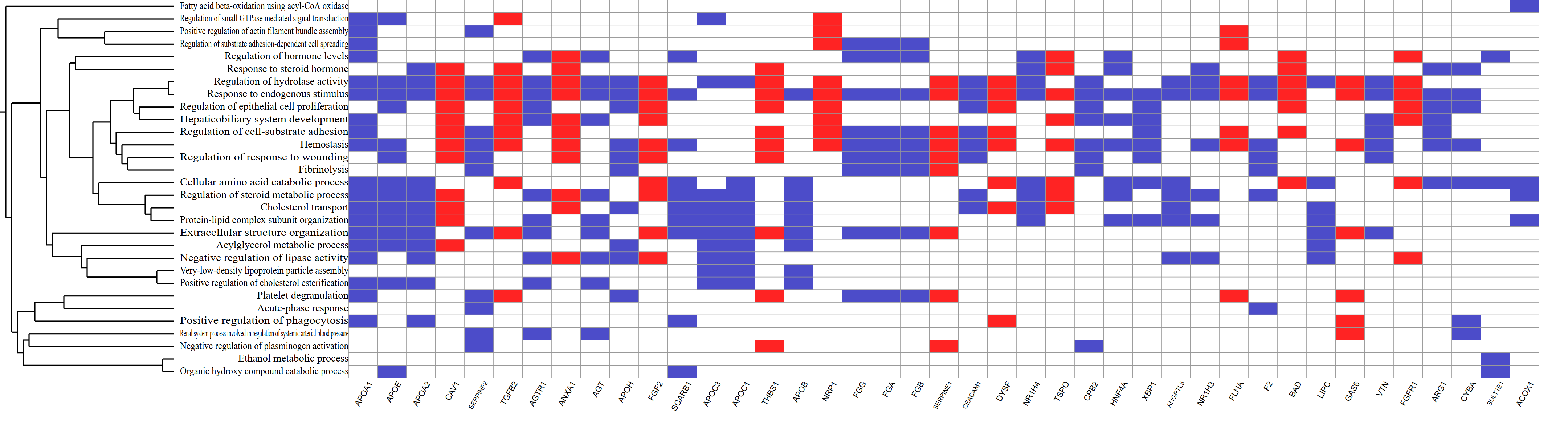

Supplement: Supplementary file 1 [file genes-11-00623-s001.zip › Figure S8.png]

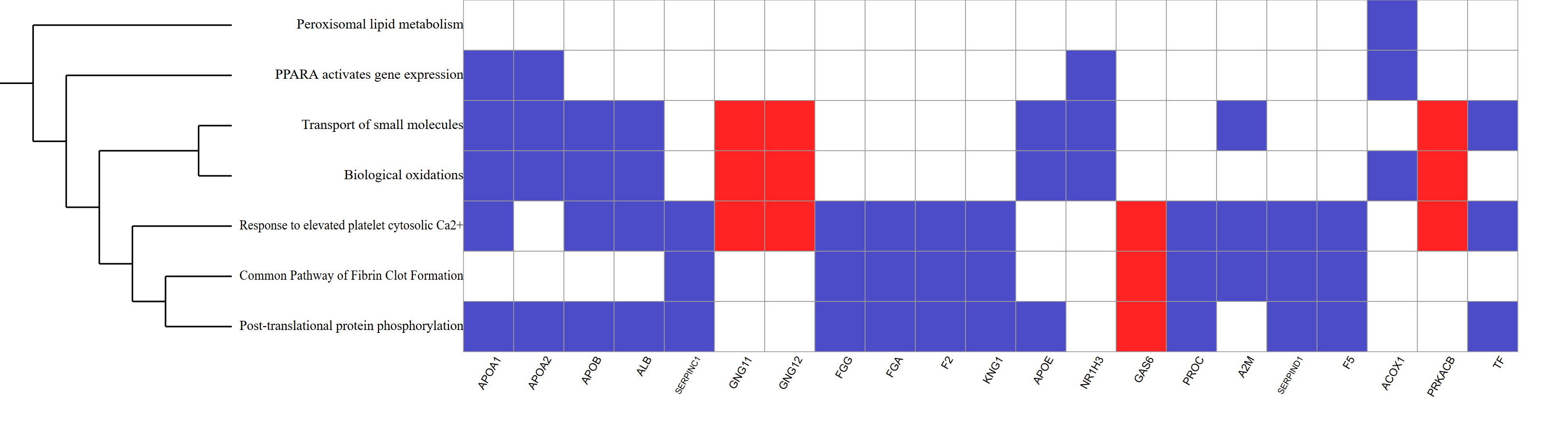

Supplement: Supplementary file 1 [file genes-11-00623-s001.zip › Figure S9.png]

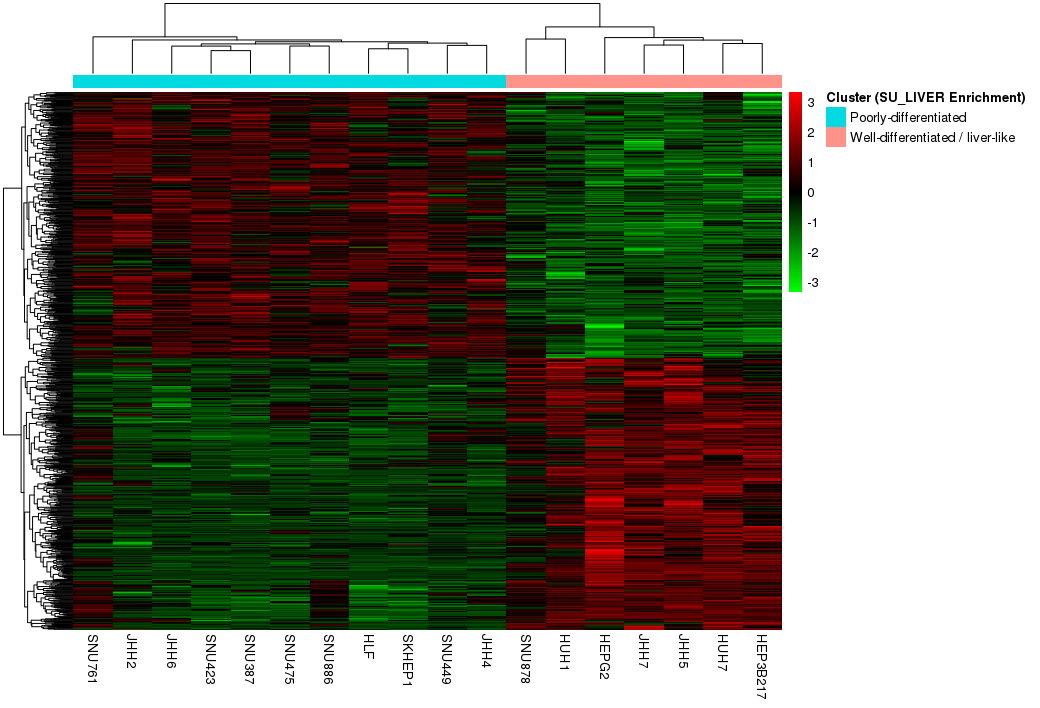

Supplement: Supplementary file 1 [file genes-11-00623-s001.zip › Figure S6.tiff]

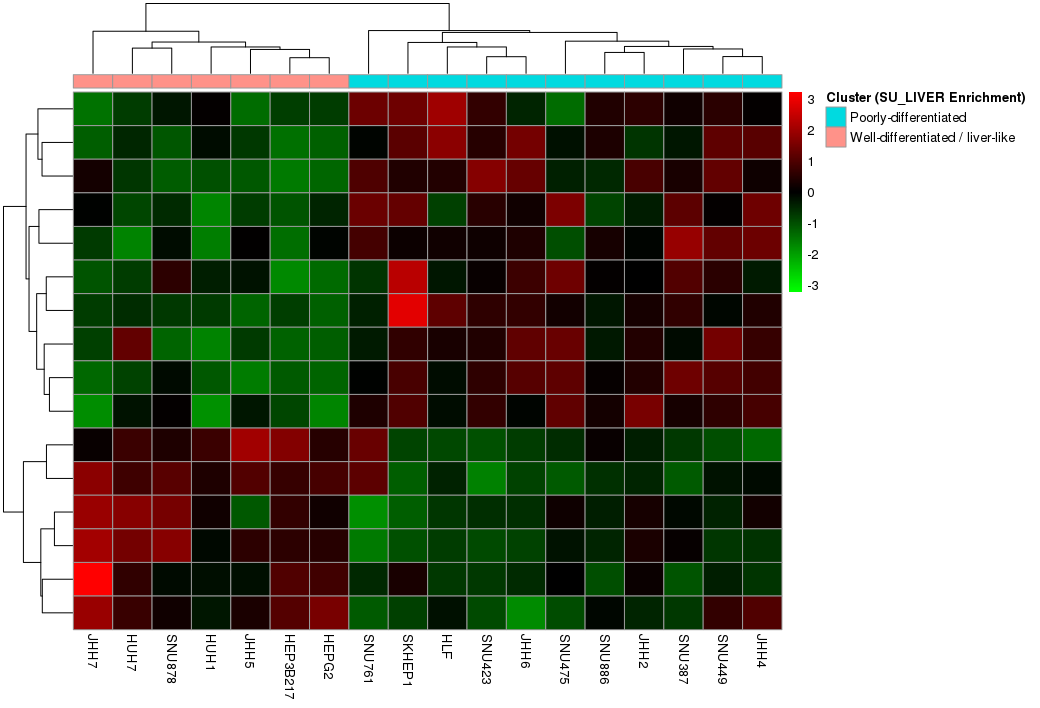

Supplement: Supplementary file 1 [file genes-11-00623-s001.zip › Figure S7.tiff]
